# Redox‐Mediated Electrochemical Regeneration of Spent LiFePO_4_ Battery Cathodes

**DOI:** 10.1002/anie.202520213

**Published:** 2026-02-10

**Authors:** Deok‐Ho Roh, Dayun Jung, James B. Gerken, Jesse J. Martinez, Eric Kazyak, Shannon S. Stahl

**Affiliations:** ^1^ Department of Chemistry University of Wisconsin−Madison Madison Wisconsin USA; ^2^ Department of Mechanical Engineering University of Wisconsin−Madison Madison Wisconsin USA

**Keywords:** Electrochemical Relithiation, LiFePO_4_, Lithium‐Ion Battery Recycling, Mössbauer Spectroscopy, Redox‐Mediated Electrochemistry

## Abstract

Direct recycling of lithium‐ion battery cathodes offers considerable appeal over metallurgical approaches. Here, we demonstrate a mediated electrochemical method for direct regeneration of degraded LiFePO_4_ (LFP). The approach uses a redox mediator, iron propylenediamine tetraacetate, that undergoes electrochemical reduction and is circulated through an external reservoir, where it supplies the electrons needed to regenerate LFP in the presence of Li^+^ ions derived from LiOH oxidation. Rapid outer‐sphere electron transfer is observed from the mediator to the degraded LFP material. This feature, together with good aqueous solubility of the mediator (0.3 M), supports current densities up to 100 mA/cm^2^, and this electrochemical recycling process is demonstrated on 100 g scale. ^57^Fe Mössbauer spectroscopy is used to monitor the correction of structural defects in the degraded LFP, providing the basis for regeneration of LFP that matches the electrochemical performance of pristine LFP.

## Graphical abstract

This work demonstrates a redox‐mediated electrochemical method for direct recycling of spent LiFePO_4_ battery cathodes. A water‐soluble mediator, iron propylenediamine tetraacetate (Fe‐PDTA), transports electrons into an off‐electrode reservoir where it drives compositional and structural repair of degraded LiFePO_4_. This process achieves current densities up to 100 mA/cm^2^ and regenerates LiFePO_4_ with performance comparable to pristine material.

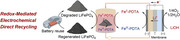

## 1 Introduction

**FIGURE 1:**
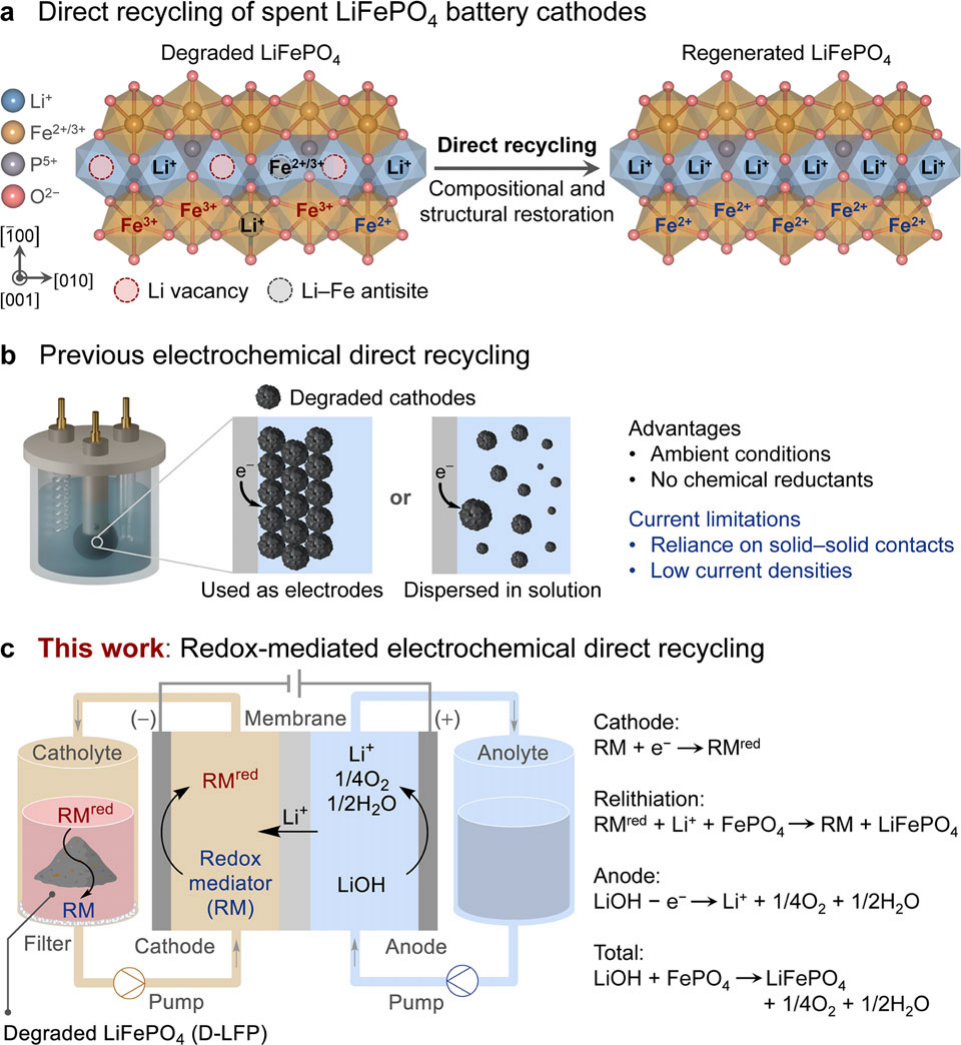
(a) Schematic of degraded and regenerated structures of LiFePO_4_ (LFP). (b) Previous electrochemical methods for direct recycling of LFP. (c) Schematic of redox‐mediated electrochemical method for direct recycling of LFP.

Recycling spent lithium‐ion batteries (LIBs) is essential to mitigate the environmental impact of battery disposal and extraction of raw materials for their production [1, 2, 3, 4, 5]. Traditional metallurgical recycling methods, which convert spent LIB cathodes into their elemental components, consume significant energy and raise environmental concerns [5, 6, 7]. LiFePO_4_ (LFP) is emerging as a dominant cathode material and will be a major component in end‐of‐life LIB waste [8, 9, 10]. The low‐cost elements present in LFP demand an economically viable recycling strategy that differs from conventional methods. Direct recycling methods that regenerate cathode materials without returning them to their elemental components offer a potential solution [5, 11, 12, 13]. Degradation of LFP is mainly attributed to lithium‐ion loss that leads to Li vacancies and oxidation of Fe^II^ to Fe^III^, causing an irreversible phase transition from LFP to FePO_4_ and formation of Li–Fe antisite defects (Figure 1a) [14, 15, 16, 17]. A reductive environment can promote relithiation, in addition to restoring the original LFP structure [12, 13, 14, 18]. Existing methods for direct recycling include solid‐state sintering [19, 20, 21], hydrothermal [22, 23, 24, 25], eutectic solvent [26, 27], and chemical [28, 29, 30, 31, 32, 33, 34, 35] strategies that feature elevated temperatures, pressures, and/or stoichiometric chemical reductants [5]. An electrochemical approach could offer improved sustainability by driving relithiation under moderate conditions and enabling the use of reductants that avoid problematic waste [36, 37, 38, 39, 40, 41, 42].

Several relevant precedents provide a foundation for the electrochemical strategy explored herein. Previous electrochemical recycling methods have used degraded cathodes as electrodes or dispersed cathode materials in the electrolyte. Electron transfer in these systems relies on solid‐solid contact between the electrode and insoluble cathode particles (Figure 1b), contributing to relatively low current densities (<2.5 mA/cm^2^, 10 mA/g_LFP_) [36, 37, 38, 39, 40, 41, 42]. Redox mediator (RM) strategies have been investigated to bypass these solid‐solid contact limitations. In one formulation, soluble RMs have been used to shuttle electrons and Li^+^ ions from Li metal to degraded cathode materials [30, 31, 32, 33, 34, 35]. This approach appears quite promising owing to its near‐ideal atom economy; however, it requires the use of aprotic organic solvents that are compatible with Li metal. A complementary approach pairs RMs with electrochemistry and takes inspiration from redox‐targeting flow batteries, where the RMs are used to shuttle electrons between an electrode and solid energy storage materials (*e.g*., LFP) [43, 44, 45, 46]. Application of a similar concept could support electrochemical recycling of battery materials and has been reported for Li^+^ extraction from spent LFP [47, 48, 49]. An initial demonstration of this concept was reported recently using anthraquinone‐2,7‐disulfonic lithium salt as a RM to promote reduction/relithiation of spent LFP cathodes, albeit still at low current density (10 mA/cm^2^ on 1 g scale) [50].

Here, we report a highly effective redox‐mediated electrochemical strategy for direct recycling of LFP (Figure 1c). A water‐soluble and electrochemically regenerable mediator shuttles electrons between an electrode and the degraded LFP in an off‐electrode reservoir, inducing LFP regeneration. The Li^+^ ions and electrons needed for LFP regeneration are supplied by the oxygen evolution reaction (OER) in a LiOH‐containing electrolyte. The iron chelate, Fe‐PDTA (PDTA = propylenediamine tetraacetate), is identified as an energy‐efficient mediator that enables rapid outer‐sphere electron transfer from Fe^II^‐PDTA to the degraded LFP at a low overpotential with respect to the LFP. This rapid redox behavior and the good water solubility of Fe‐PDTA contribute to electrochemical current densities up to 100 mA/cm^2^ and 40 mA/g_LFP_, and the process is demonstrated on 100 g scale. The electrochemically reduced Fe^II^‐PDTA generates a reductive environment that induces relithiation and correction of Li–Fe antisite defects, evident from powder X‐ray diffraction (PXRD) analysis and other methods. ^57^Fe Mössbauer spectroscopy provides an effective and sensitive probe of LFP regeneration by detecting all Fe species within bulk LFP, revealing electrochemically inactive Fe^III^ species that are not evident by other methods but need to be corrected to access near‐pristine LFP performance. Techno‐economic and environmental analysis support the economic viability of this strategy and reveal further opportunities to reduce the process cost and environmental impact.

## 2 Results and Discussion

The overall strategy of redox‐mediated electrochemical direct recycling is illustrated in Figure 1c. The RM is reduced to RM^red^ at the cathode and then pumped through the catholyte reservoir containing degraded LFP (D‐LFP). RM^red^ reduces Fe^III^ to Fe^II^ in D‐LFP *via* outer‐sphere electron transfer, coupled to Li^+^ insertion into the solid‐state D‐LFP to provide charge balance, resulting in phase conversion from FePO_4_ to LFP phase (*i.e*., relithiation). The oxidized RM recirculates to the cathode and repeats the process. Charge balance is achieved through oxygen evolution at the anode, which contains LiOH electrolyte. Li^+^ ions migrate from the anolyte to the catholyte through a cation‐exchange membrane (CEM; Nafion in the present study). The entire process consumes only electricity and LiOH and generates only H_2_O and O_2_ as byproducts.

### 2.1 Redox Mediator Selection

The direct recycling method considered here resembles redox‐targeting flow batteries [43, 44, 45, 46, 51, 52], as noted above; however, the criteria for selection of the optimal mediator in these two applications are different. In the flow batteries, the mediator should have a redox potential nearly identical to that of solid‐state material (*e.g*., LFP) to minimize cell voltage loss and operate in both charging and discharging processes. In the present direct recycling method, the optimal mediator will have overpotential with respect to the LFP to support full reduction and relithiation of the material.

**FIGURE 2:**
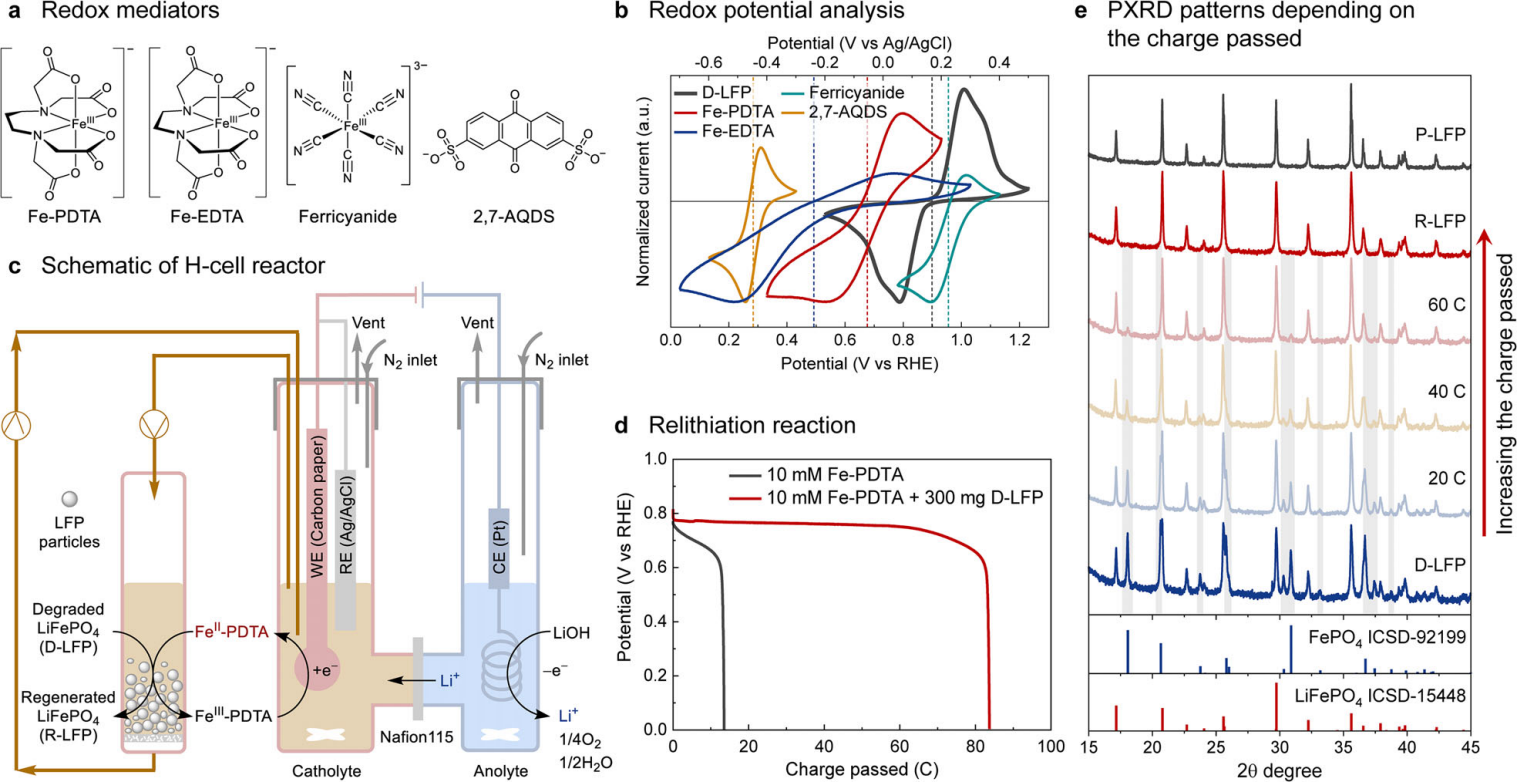
(a) Chemical structures of redox mediators; PDTA = propylenediamine tetraacetate, EDTA = ethylenediamine tetraacetate, 2,7‐AQDS = 2,7‐anthraquinone disulfonic acid. (b) Cyclic voltammetry (CV) curves of redox mediators (10 mM) and degraded LiFePO_4_ (D‐LFP) at a scan rate of 1.0 mV/s in a three‐electrode system; 1 M borate, pH 9. Vertical dashed lines indicate the half‐wave potentials (*E*_1/2_). (c) Schematic of an H‐cell interfaced in a recirculating flow configuration with a reservoir containing D‐LFP. (d) Chronopotentiometry curves of Fe‐PDTA with and without D‐LFP at −1 mA/cm^2^. (e) Powder X‐ray diffraction patterns (PXRD) of LFP at different quantities of charge passed. The gray‐shaded region indicates heterosite FePO_4_ peaks.

To initiate our studies, D‐LFP was isolated from spent commercial LFP cells with 45–60% remaining capacity (Figures S1 and S2). The redox properties of various water‐soluble mediators were then analyzed by cyclic voltammetry (CV), and their potentials were compared to that of D‐LFP (Figure 2a and b). Among the candidates, an iron complex of propylenediamine tetraacetate (Fe‐PDTA) shows several appealing characteristics. It is highly water‐soluble (up to 0.35 M), low‐cost, and exhibits reversible CV behavior with a redox potential sufficient to promote full reduction of the D‐LFP with only modest overpotential (Figure 2b) [53, 54, 55]. Specifically, Fe‐PDTA has a redox potential of 0.68 V while (D‐)LFP has a potential of 0.90 V (all potentials reported versus the reversible hydrogen electrode, RHE), corresponding to an overpotential of only 0.22 V. Ferricyanide, with a redox potential of 0.96 V, is thermodynamically unfavorable for D‐LFP reduction. Another iron complex, iron ethylenediamine tetraacetate (Fe‐EDTA), has a potential of 0.49 V but shows quasi‐reversible redox behavior, likely associated with *μ*‐oxo dimer formation [56]. 2,7‐Anthraquinone disulfonic acid (2,7‐AQDS) exhibits reversible electrochemistry, but its redox potential of 0.28 V results in a relatively large overpotential of 0.62 V with respect to LFP.

The kinetics of D‐LFP reduction and Li^+^ charging in the presence of mediator were investigated by probing “chemical” regeneration of D‐LFP with excess reduced mediator and Li^+^ (approx. 4 equiv. Fe^II^‐PDTA, Fe^II^‐EDTA, or anthrahydroquinone‐2,7‐disulfonate (AHQDS), the reduced form of AQDS, relative to the estimated Li^+^ vacancies). The time courses were monitored by performing a series of reactions that were stopped at different times to analyze the relative fraction of LFP phase in the bulk material by PXRD (Figures S3 and S4). The time‐course data show that the LFP phase rapidly increases from 55% to >90% within the first 3 min for all three mediators, with slightly higher rates for more reducing mediators. Full regeneration of the remaining 10% of the LFP phase requires somewhat longer times and shows minimal dependence on the mediator identity. The latter process is attributed to slower Li^+^ diffusion through the bulk LFP (estimated to be 10^−14^ to 10^−16^ cm^2^/s [57]). Based on these kinetic data, the lower overpotential of Fe‐PDTA motivated its selection as the mediator for subsequent LFP regeneration experiments.

### 2.2 Initial Tests of Mediated Electrochemical Regeneration of Degraded LiFePO_4_

Initial relithiation tests were conducted in an H‐cell (Figures 2c and S5). As shown in Figure 2d, the charge passed increased from 13.5 C for Fe‐PDTA alone to 83.7 C in the presence of D‐LFP, consistent with reduction and relithiation of D‐LFP promoted by electrochemical regeneration of the mediator. The Faradaic efficiency of the process is nearly 100% on the basis of batch control experiments that probed the total charge needed for complete regeneration of D‐LFP (see Figure S6 for details). After complete relithiation and formation of regenerated LFP (R‐LFP), the potential sharply decreased and the reaction self‐terminated at the target potential, preventing overlithiation.

PXRD confirmed phase restoration of D‐LFP to that resembling pristine LFP (P‐LFP). Figure 2e shows the evolution of the LFP PXRD patterns at different levels of charge passed. D‐LFP exhibits additional peaks at 18° and 32°, corresponding to heterosite FePO_4_ [58]. With increased passage of charge, the peak intensities of FePO_4_ gradually decreased and eventually disappeared, and the R‐LFP exhibited a single LFP phase that matched P‐LFP. The correlation between LFP phase restoration and quantity of charge passed indicates that relithiation is driven by reductive electrochemical cycling of the Fe‐PDTA mediator. As confirmed by further analysis below, this process also leads to correction of the Li–Fe antisite defects present in the D‐LFP.

### 2.3 Closed‐Loop Regeneration

**FIGURE 3:**
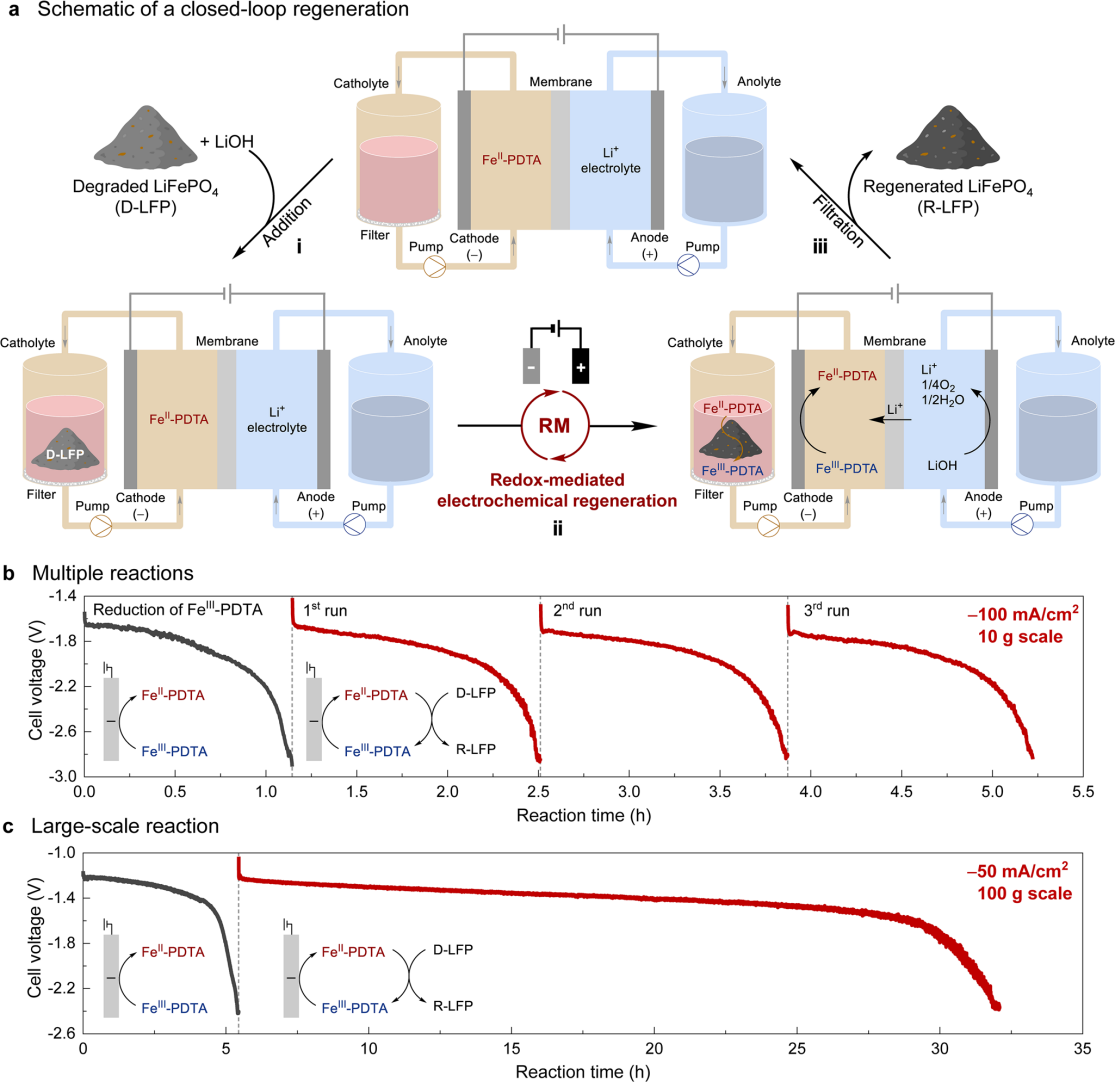
(a) Schematic of the closed‐loop regeneration process: (i) addition of degraded LiFePO_4_ (D‐LFP) and LiOH to the catholyte and anolyte, respectively, (ii) mediated electrochemical relithiation of D‐LFP; (iii) collection of regenerated LFP (R‐LFP). Chronopotentiometry traces obtained during (b) sequential 10 g scale reactions at −100 mA/cm^2^ (the initial black trace reflects charging of the mediator in the absence of D‐LFP) and (c) a 100 g scale reaction at −50 mA/cm^2^. No special effort was applied to maintain anaerobic conditions in the large‐scale experiments.

Efforts to evaluate a closed‐loop regeneration process were initiated by adding solid D‐LFP to a reservoir containing the catholyte with Fe^II^‐PDTA (0.3 M) and charging the anodic reservoir with LiOH electrolyte (step i, Figure 3a). LFP regeneration is achieved by reducing Fe^III^‐PDTA to Fe^II^‐PDTA at the cathode and recirculating the catholyte solution over the D‐LFP powder to promote regeneration of the material in the external reservoir (step ii). Li^+^ ions are generated by oxygen evolution at the anode and transported through the CEM. Upon full regeneration, the R‐LFP is collected by filtration and the catholyte containing Fe^II^‐PDTA is reused for subsequent cycles (step iii). This system is readily scaled by increasing the size of the D‐LFP reservoir and solution volumes (Figure S7), and an electrolysis flow cell was employed for larger‐scale regeneration tests (Figures S8 and S9). All large‐scale experiments were conducted without anaerobic control. In addition, the high current densities with Fe‐PDTA are directly related to the fast outer‐sphere electron transfer from Fe^II^‐PDTA to D‐LFP (Figure S3). Specifically, each time the reduced mediator passes through the reservoir containing D‐LFP, it will undergo efficient electron transfer, regenerating Fe^III^‐PDTA prior to flowing past the electrode. The high concentration of Fe^III^‐PDTA at the electrode surface accounts for the high observed current density. The good solubility of Fe‐PDTA (0.3 M) and the fast electron transfer from Fe^II^‐PDTA to D‐LFP support operation at current densities up to −100 mA/cm^2^ (Figure S10), reflecting at least an order‐of‐magnitude higher rate than previous electrochemical direct recycling methods [36, 37, 38, 39, 40, 41, 42, 50].

Representative results in Figure 3b feature a series of 10 g‐scale regeneration reactions, performed at −100 mA/cm^2^. Initial reduction of Fe^III^‐PDTA to Fe^II^‐PDTA was performed in the absence of D‐LFP (black trace, Figure 3b). With D‐LFP in the catholyte reservoir during subsequent stages, the electrolysis current reflects reduction of Fe^III^‐PDTA generated during chemical relithiation of D‐LFP in the reservoir. In these stages, the initial cell voltage was −1.7 V and gradually progressed to −2.8 V as relithiation reached completion (Figure 3b). This voltage contained an OER overpotential that is approximately 2.3 times larger than that required for Fe‐PDTA reduction (Figure S11). It should be noted that Ni foam was used as the anode for simplicity, without any effort to optimize the OER. Future efforts could improve energy efficiency by using better OER electrocatalysts, such as Ni–Fe oxides [59, 60]. After each stage, the R‐LFP was collected by filtration, a new batch of D‐LFP was added to the catholyte reservoir, and LiOH was replenished in the anolyte, based on the amount consumed. Each of the regeneration steps exhibited nearly identical behavior, suggesting no obvious degradation of the Fe‐PDTA mediator over multiple cycles (Figures 3b and S12). In addition, no oxidation of the Fe^II^‐PDTA catholyte was observed during handling in ambient conditions across multiple cycles because its oxidation is sufficiently slow (Figures S12 and S13). Although Fe^II^‐PDTA is potentially capable of oxidation by O_2_, it reacts very slowly, with less than 0.5% of Fe^II^‐PDTA undergoing oxidation when exposed to ambient air over 60 min. Even when bubbling 1 atm O_2_ through a stirred solution of Fe^II^‐PDTA, only 2.8% Fe^III^‐PDTA was formed. This air‐insensitivity likely arises from inhibition of inner‐sphere reaction with O_2_ by PDTA chelation of Fe [61] and has important practical implications as it enables operation of the process without requiring anaerobic conditions. To validate the scalability of the process, we used a larger catholyte reservoir and performed the reaction on 100 g scale (Figure 3c). The characterization data of the larger scale material was very similar to those of 10 g scale R‐LFP (see Figure S14 for details).

The time scale of the mediated electrochemical process described herein appears to be largely controlled by the current density associated with electrochemical regeneration of the mediator. The latter point is evident from comparison of the voltage/time traces in Figure 3b and c, where the steady‐state voltage is relatively stable throughout much of the time course and closely matches the initial voltage observed for reduction of the mediator in the absence of D‐LFP. This observation suggests that the majority of the Fe‐PDTA mediator that flows past the cathode is in the oxidized state, having successfully transferred its electrons to the D‐LFP in the reservoir. If the mediator concentration is maintained at 0.3 M (close to the mediator solubility limit in water), the total time required to regenerate the D‐LFP material may be estimated from the current density (mA/cm^2^), the surface area of the electrode (cm^2^), and the quantity and degradation state of the D‐LFP material that needs to be regenerated (*i.e*., the total charge required). The rate may be slowed somewhat by the rate of solid‐state Li^+^ diffusion within the LFP at later stages of D‐LFP regeneration (*i.e*., at >90% LFP regeneration; see Figures S3 and S4 for additional context), but this process is expected to be only a secondary factor in the overall time needed for D‐LFP regeneration.

Fe‐PDTA crossover and cycling tests were conducted to assess the long‐term stability of the system. For example, the crossover Fe concentration in the anolyte from 0.3 M Fe‐PDTA catholyte reservoir was monitored over time under operation at a constant current density of −20 mA/cm^2^ (Figure S15). The resulting permeation rate of 4.43 × 10^−13^ mol/cm^2^.s corresponds to 130 ppm crossover of Fe‐PDTA into the anolyte over 1000 h of operation under the same 100 g scale conditions. This low crossover rate likely reflects the electro‐osmotic drag associated with Li^+^ cation transport through the membrane in the opposite direction (from the anolyte to the catholyte) during electrolysis, in addition to electrostatic exclusion of the anionic Fe‐PDTA complex by the cation‐selective membrane. In short, crossover of Fe‐PDTA will not limit the long‐term stability of the system. The cycling test of Fe‐PDTA was performed using a symmetrical redox flow‐battery approach at a constant current density of 30 mA/cm^2^ (Figure S16) [62]. Fe‐PDTA maintained >95% capacity for over 75 cycles (86 h), beyond which degradation started to become evident, resulting in 86.2% capacity retention of Fe‐PDTA after 100 cycles and 110 h. These cycling data suggest that the Fe‐PDTA will be capable of undergoing approximately 75 cycles (formally corresponding to the mediator “turnover number”) before degradation will be observed. This parameter may be combined with techno‐economic analysis (TEA) to assess the long‐term performance of the mediator. As elaborated below, the TEA incorporates a conservative estimate of 10% replenishment of the mediator/electrolyte solution with every cycle through the reservoir. With the indicated cycling stability and rate of mediator/electrolyte replacement, the process will never accumulate significant quantities of degraded mediator.

### 2.4 Compositional and Structural Characterization of Regenerated LiFePO_4_

**FIGURE 4:**
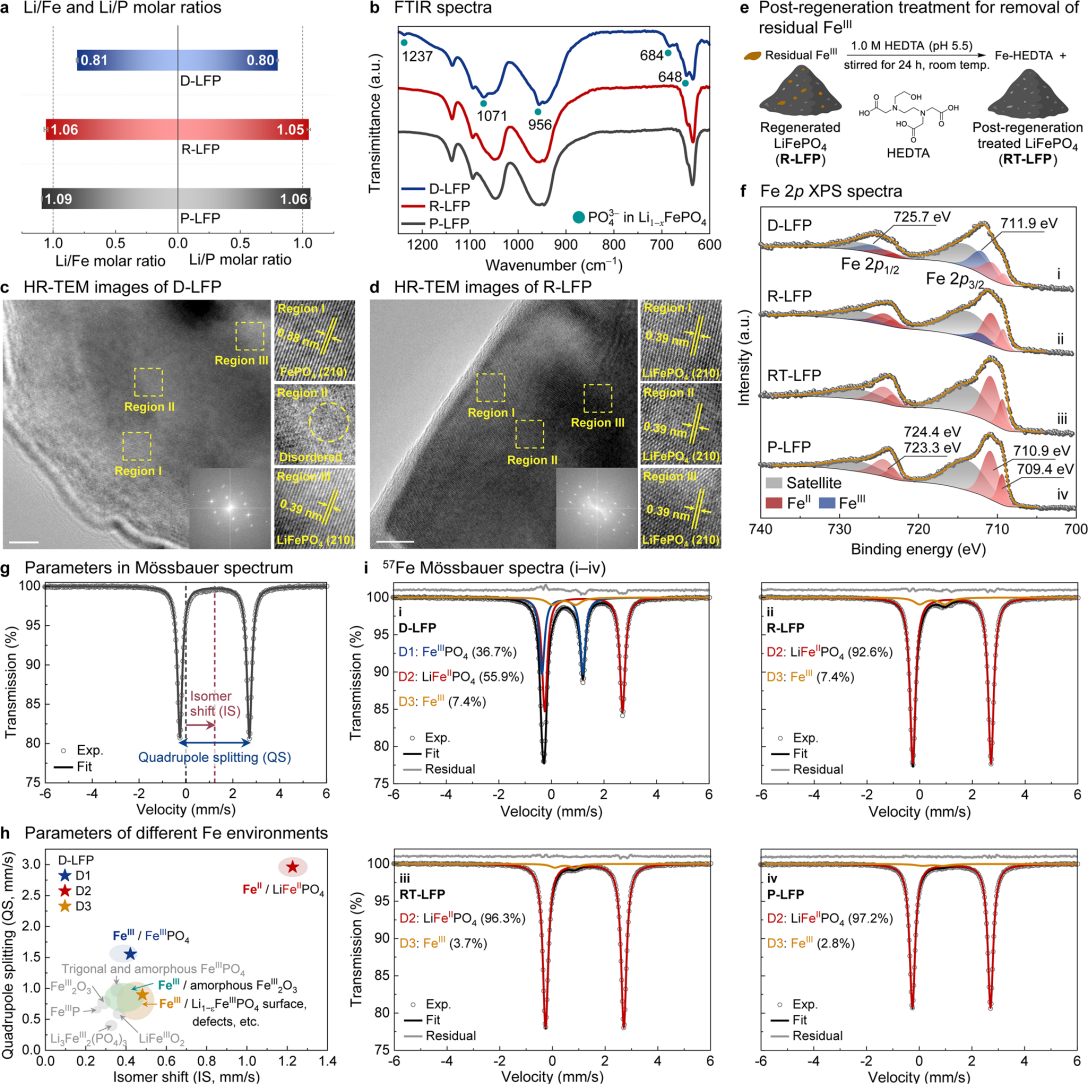
(a) Li/Fe and Li/P molar ratios in degraded, regenerated, and pristine LiFePO_4_ (D‐, R‐, and P‐LFP). (b) FTIR spectra. High‐resolution transmission electron microscopy (HR‐TEM) images of (c) D‐LFP and (d) R‐LFP with corresponding fast Fourier transform patterns. (e) Post‐regeneration treatment. (f) Fe 2*p* X‐ray photoelectron spectra (XPS). (g) Schematic illustration of the isomer shift and quadrupole splitting in a Mössbauer spectrum. The experimental data and fitted curve correspond to P‐LFP with Fe^III^ contributions excluded. (h) Quadrupole splitting versus isomer shift for different Fe environments. (i) ^57^Fe Mössbauer spectra (295 K). Fitting parameters are summarized in Figure S19.

The compositional and structural restoration of R‐LFP was investigated by spectroscopic and microscopic analyses. Inductively coupled plasma‐optical emission spectroscopy (ICP‐OES) confirmed Li replenishment, as the Li/Fe and Li/P molar ratios increased from 0.81 and 0.80 in D‐LFP to 1.06 and 1.05, respectively, in R‐LFP, the latter being comparable to those of P‐LFP (Figure 4a). The Li/Fe and Li/P molar ratios in D‐LFP are higher than expected from the extent of capacity loss in the spent cathodes. This apparent discrepancy, however, reflects the presence of solid‐electrolyte interphase Li species (*e.g*., LiF), which are evident from F 1*s* XPS analysis of the material (Figure S17). Fourier transform infrared spectroscopy (FTIR) showed structural restoration, as the PO_4_^3−^ vibrational peaks associated with Li vacancies in D‐LFP disappeared (cyan dots in Figure 4b), and the spectrum of R‐LFP closely aligned with that of P‐LFP [63, 64]. High‐resolution transmission electron microscopy (HR‐TEM) revealed the atomic‐level microstructures of D‐LFP and R‐LFP from the surface to the interior (Figure 4c and d). In D‐LFP, a carbon coating layer (2–4 nm) remained on the LFP surface, and particle cracking was not observed in the measurements, indicating no severe particle‐scale structural degradation. However, at the atomic scale, the FePO_4_ and LFP phases coexisted with disordered regions. Region I at the surface showed 0.38 nm lattice spacing of the FePO_4_ (210) plane. Region II showed disordered areas due to the coexistence of FePO_4_ and LFP phases. Region III in the interior exhibited 0.39 nm lattice spacing, corresponding to the LFP (210) plane [21, 23, 24]. After regeneration, R‐LFP retained the carbon coating layer and showed uniform 0.39 nm lattice spacing across all regions, indicating restoration to a single LFP phase. Reordering of Li–Fe antisite defects was estimated using Rietveld refinement of PXRD patterns (Figure S18). D‐LFP showed 2.0 ± 0.4% antisite defects, which decreased to 1.5 ± 0.2% in R‐LFP as the reductive environment reduced Fe^III^ to Fe^II^ and decreased electrostatic repulsion between Fe atoms, enabling antisite reordering [18, 22, 29, 31]. Collectively, these data show that mediated regeneration not only resolves Li vacancy defects but also induces the correction of Li–Fe antisite defects in LFP.

### 2.5 Post‐Regeneration Treatment of LiFePO_4_

The change in the Fe valence state of LFP at the surface and in the bulk was investigated using X‐ray photoelectron spectroscopy (XPS), together with ^57^Fe Mössbauer spectroscopy (Figures 4e–i and S19). Mössbauer spectroscopy enables quantitative detection of all Fe species in the bulk material [65]. Figure 4g illustrates the Mössbauer doublet spectrum associated with P‐LFP, which has an isomer shift (IS) of 1.23 mm/s and a quadrupole splitting (QS) of 2.96 mm/s. The IS provides insight into the electron density at the Fe nucleus and often correlates with the Fe oxidation state. The QS arises from an inhomogeneous electric field at the nucleus and provides information about the local electronic structure and lattice environment. The IS and QS hyperfine parameters allow distinction of different Fe species within the bulk material (Figure 4h), and the area of each spectral component is proportional to the relative fraction of each of the Fe species. XPS is a surface‐specific analytical technique and it provides valuable qualitative insight into different Fe species in different electronic environments within the material [66, 67]. The data obtained from both of these methods reveal the presence of residual Fe^III^ in R‐LFP.

It was possible to eliminate residual Fe^III^ in R‐LFP by a post‐regeneration treatment, consisting of stirring a slurry of R‐LFP in 1.0 M hydroxyethyl ethylenediamine triacetate (HEDTA) solution under ambient conditions (Figure 4e). HEDTA chelates residual Fe^III^ in R‐LFP, facilitating its removal by dissolution [20]. Compositional and structural characterization data from the treated R‐LFP (RT‐LFP) are summarized in Figure S20, with the most notable content associated with changes in the Fe valence state (Figures 4f and 4i). D‐LFP exhibited Fe^III^ XPS peaks at 711.9 and 725.7 eV, associated with FePO_4_, and Fe^II^ XPS peaks at 709–711 and 723–724 eV corresponding to LFP (Figure 4f–i) [66, 67]. The Mössbauer spectrum of D‐LFP was resolved into three doublet peaks (D1–D3) in Figure 4i–i; D1 is assigned to FePO_4_, D2 to LFP, and D3 to Fe^III^ impurities. The 0.42 mm/s IS of D1 is associated with an Fe^III^ oxidation state, and the QS value of 1.55 mm/s is consistent with FePO_4_ [68]. For D2, the 1.23 mm/s IS aligns with an Fe^II^ oxidation state, and it has a characteristically large QS value of 2.96 mm/s due to the asymmetric octahedral FeO_6_ environment in LFP [68, 69, 70, 71, 72, 73, 74, 75]. These IS and QS values for D1 and D2 are consistent with reported literature values, as shown in Figure 4h. D3 is assigned to Fe^III^ impurities (Figure 4h), as the typical IS value for Fe^III^ is 0.4–0.5 mm/s [76], and its broad full width at half maximum (FWHM: >0.3 mm/s) suggests contributions from amorphous or nanoparticulate phases, surface or structural defects in LFP, and/or multiple Fe^III^ components (*e.g*., iron oxides) [70, 71, 72, 73, 74, 75, 77]. The area contributions of these different components correspond to 36.7% FePO_4_ and 7.4% Fe^III^ impurities in D‐LFP, reflecting significant oxidative degradation. After regeneration, the Mössbauer spectrum was consistent with conversion of FePO_4_ into LFP (Figure 4i–ii). Fe^II^ peaks were also dominant in the XPS data (Figure 4f–ii). Small quantities of Fe^III^, however, were still evident in R‐LFP, according to the Mössbauer and XPS data. Post‐regeneration treatment by the HEDTA chelate led to disappearance of this Fe^III^ feature in the XPS data, resulting in a spectrum that closely resembled P‐LFP (Figure 4f–iii,iv). Mössbauer spectroscopy is more sensitive and showed a reduction of the Fe^III^ content to 3.7% in RT‐LFP from 7.4% in R‐LFP, close to the 2.8% Fe^III^ content in P‐LFP (Figure 4i–iii,iv). These results highlight the value of Mössbauer spectroscopy as an effective and sensitive probe of LFP regeneration through its ability to reveal structural degradation into amorphous or disordered phases that are not clearly detectable by other methods. This analysis provided the basis for the post‐regeneration treatment that accesses pristine‐level LFP performance, as elaborated below.

### 2.6 Electrochemical Performance Evaluation of LiFePO_4_ Materials

**FIGURE 5:**
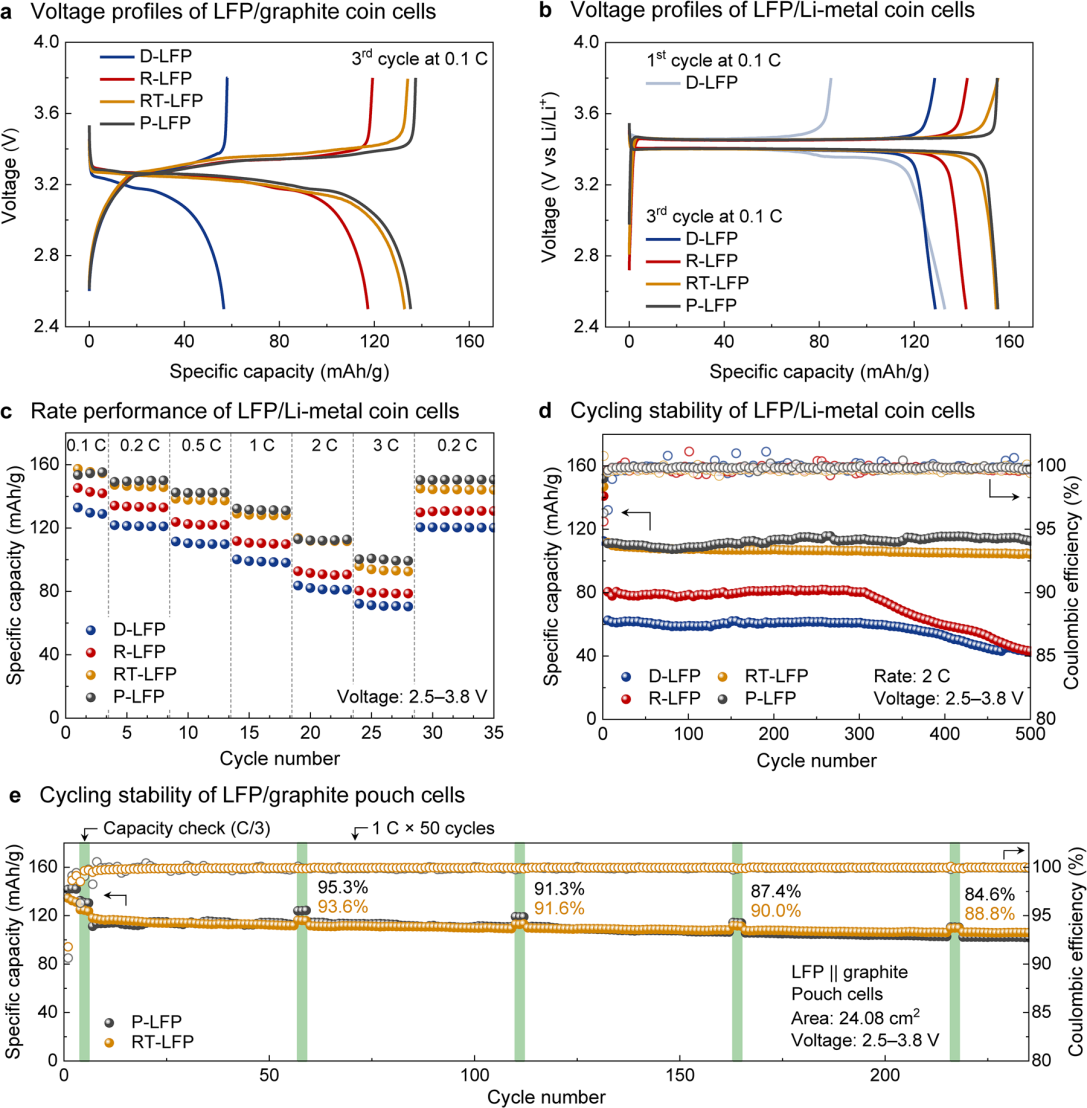
Voltage profiles of (a) LFP/graphite and (b) LFP/Li‐metal coin cells at 0.1 C. (c) Rate performance and (d) cycling stability of LFP/Li‐metal coin cells. (e) Cycling stability of LFP/graphite pouch cells.

The electrochemical performance of the different LFP materials was then evaluated using both LFP/graphite and LFP/Li‐metal cell configurations (Figure 5). Voltage profiles of LFP/graphite coin cells show an enhanced capacity of 117 mAh/g for R‐LFP compared to the remaining capacity of 57 mAh/g for D‐LFP (Figure 5a). RT‐LFP showed even better capacity of 133 mAh/g, comparable to P‐LFP (135 mAh/g). Complementary data were obtained with LFP/Li‐metal coin cells (Figure 5b). D‐LFP was partially relithiated from the Li‐metal anode during the initial discharge, providing an enhanced capacity of 129 mAh/g at a rate of 0.1 C. R‐LFP still showed a higher capacity of 142 mAh/g than D‐LFP due to complete relithiation during the regeneration process. The capacity of R‐LFP remained lower than that of P‐LFP; however, the post‐regeneration treatment described above enabled the RT‐LFP to access a capacity of 155 mAh/g, matching that of P‐LFP at 0.1 C. Similar trends were evident from rate performance studies (Figures 5c and S21). At rates of 0.2, 0.5, 1, 2, and 3 C, R‐LFP exhibited higher values than D‐LFP, but lower than RT‐LFP, which performed similarly to P‐LFP. Cycling stability studies (Figure 5d) showed that capacity faded with D‐LFP and R‐LFP after 300 cycles at 2 C, whereas RT‐LFP maintained 94.8% retention after 500 cycles. Finally, LFP/graphite pouch cells were assembled and evaluated to assess performance in a more practical configuration (Figure 5e). RT‐LFP exhibited a capacity retention of 88.8% at C/3 after 200 cycles at 1 C, which is higher than the 84.8% retention observed with P‐LFP. Collectively, these results show that the RT‐LFP material derived from mediated electrochemical regeneration of D‐LFP and post‐regeneration chelation treatment to remove residual Fe^III^ from R‐LFP closely matches the electrochemical performance of P‐LFP.

### 2.7 Techno‐Economic and Environmental Analysis

**FIGURE 6:**
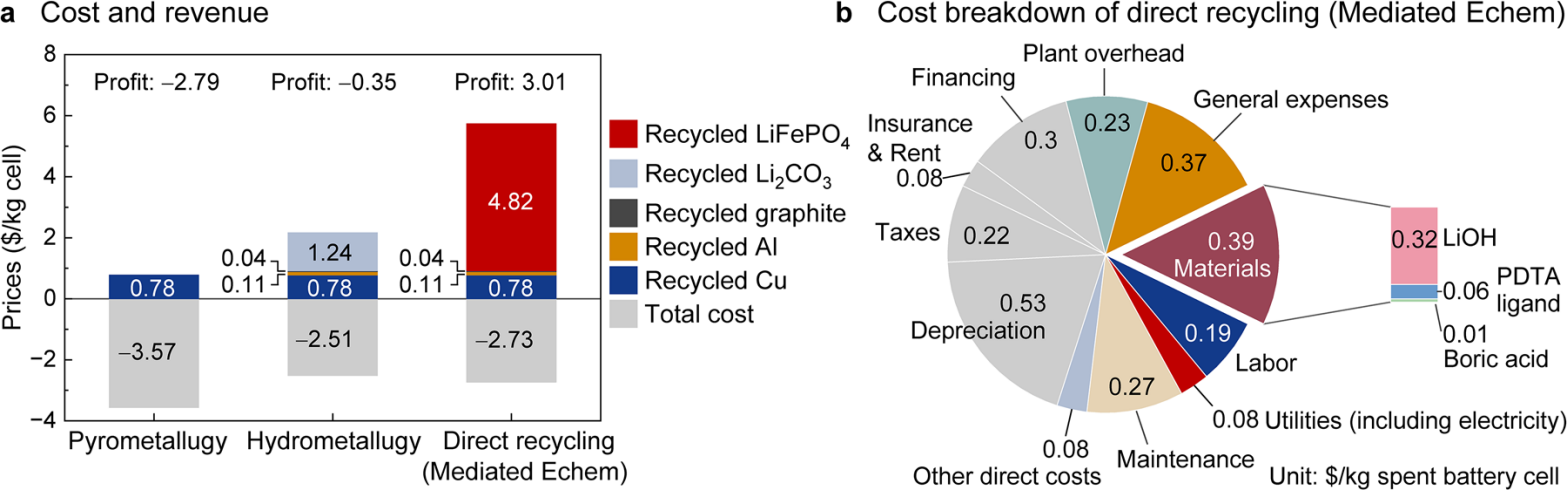
Techno‐economic assessment of the redox‐mediated electrochemical direct recycling process (denoted as Mediated Echem) compared to pyrometallurgical and hydrometallurgical processes. (a) Cost and revenue for recycling 1 kg spent battery cells. (b) Cost breakdown of the Mediated Echem process. The category of other direct costs in the panel includes operating supplies, laboratory charges, patents, and royalties. The cost of electricity ($0.075/kg cell) is included in the utilities category.

The cost and environmental impact of the redox‐mediated electrochemical direct recycling process were evaluated using the EverBatt model [78] and compared with similar analyses of pyrometallurgical and hydrometallurgical recycling processes (Figures 6 and S22–S24). Consistent with previous reports [20, 21, 22, 27, 28, 29, 30, 31, 34], the EverBatt‐based techno‐economic assessment indicates that direct recycling represents an economically viable approach for LFP recycling, and the present mediated electrochemical process is projected to achieve a profit of $3.01/kg spent battery cell. Traditional metallurgical processes are not expected to be economically viable due to the low value of the recycled products. Other direct recycling methods have been reported [19, 20, 21, 22, 23, 24, 25, 26, 27, 28, 29, 30, 31, 32, 33, 34, 35, 36, 37, 38, 39, 40, 41, 42, 50], and a rather close analog of the present process employs a mediator in combination with Li metal as the source of electrons and Li^+^. This approach avoids the need for an electrochemical source of the electrons [30, 31, 32, 33, 34, 35]; however, Li metal is nearly two‐fold more expensive than LiOH (estimated costs: $0.63/kg cell for Li metal and $0.32/kg cell for LiOH; Figures 6b and S22b). In addition, Li metal‐based processes require the use of an aprotic organic solvent, such as THF, dimethoxyethane, or propylene carbonate, that introduces additional costs for solvent recycling and/or replacement. The present mediated electrochemical process will benefit from a lower cost source of Li^+^ and the use of water as the solvent, raising the prospects for a more favorable cost structure (see Section 14 of the Supporting Information for additional content and discussion).

## 3 Conclusion

The redox‐mediated electrochemical process outlined herein demonstrates an effective strategy for direct recycling of spent LFP. The highly water‐soluble Fe‐PDTA mediator undergoes electrochemical reduction, and the low‐valent species provides a reductive environment capable of promoting compositional and structural repair of spent LFP in an off‐electrode reservoir. Specifically, the mediated regeneration process resolves Li vacancies and induces correction of Li–Fe antisite defects in LFP. ^57^Fe Mössbauer spectroscopy proved to be a sensitive probe of LFP degradation and provided the basis for restoration of spent LFP to near‐pristine battery performance. Practical advantages of the low‐cost Fe‐PDTA mediator include its low overpotential with respect to LFP and air‐insensitivity, reducing energy consumption and enabling operation of the process without the need for anaerobic conditions. The rapid outer‐sphere electron transfer to LFP and the good water‐solubility of Fe‐PDTA support current densities up to 100 mA/cm^2^. Techno‐economic and environmental analysis indicate that this aqueous mediated electrochemical approach offers a cost‐effective process with reduced environmental impact. These features and successful demonstration of the process on 100 g scale provide a foundation for further exploration and development as a strategy for direct recycling of Li‐ion battery materials.

## Conflicts of Interest

D.‐H. Roh, J. B. Gerken, and S. S. Stahl are inventors on a patent related to this work filed by the Wisconsin Alumni Research Foundation.

## Supporting information




**Supporting File 1**: anie71386‐sup‐0001‐SuppMat.pdf.

**Supporting File 2**: anie71386‐sup‐0002‐Data.xlsm.

## Data Availability

The data that support the findings of this study are available in the supplementary material of this article.
