# Supplementary material for: Redox‐Mediated Electrochemical Regeneration of Spent LiFePO4 Battery Cathodes
Source: Angew Chem Int Ed Engl. 2026 Feb 10;65(12):e20213. doi: 10.1002/anie.202520213 (PMC12991038; doi:10.1002/anie.202520213)
Supplement: Supplementary file 1 — Supporting File 1: anie71386‐sup‐0001‐SuppMat.pdf. [file ANIE-65-e20213-s001.pdf]

Supporting Information

©Wiley-VCH 2021

69451 Weinheim, Germany

Redox-Mediated Electrochemical Regeneration of Spent $LiFePO_4$ Battery Cathodes

Deok-Ho Roh[a], Dayun Jung[b], James B. Gerken[a], Jesse J. Martinez[a], Eric Kazyak[b]*, and Shannon S. Stahl[a]*

[a] Department of Chemistry, University of Wisconsin–Madison, Madison, Wisconsin 53706, United States

[b] Department of Mechanical Engineering, University of Wisconsin–Madison, Madison, Wisconsin 53706, United States

Table of Contents

1. **General experimental considerations......S2**
2. **Isolation of degraded $LiFePO_4$ from spent battery packs......S6**
3. **Kinetics of the reactions between degraded $LiFePO_4$ and three different mediators......S8**
4. **Small-scale regeneration of $LiFePO_4$ in an H-cell......S10**
5. **Evaluation of Faradaic efficiency......S12**
6. **Large-scale regeneration of $LiFePO_4$ in a flow cell......S13**
7. **Characterization of the 100 g scale regenerated $LiFePO_4$......S19**
8. **Fe-PDTA crossover and cycling tests......S20**
9. **Solid-electrolyte interphase species......S22**
10. **Li–Fe antisite defect analysis using Rietveld refinement of PXRD patterns......S23**
11. **^{57}Fe Mössbauer spectroscopy......S25**
12. **Characterization results of RT-LFP......S26**
13. **Voltage profiles of $LiFePO_4$/Li-metal coin cells......S27**
14. **Techno-economic and environmental analysis......S28**
15. **Characterization results of Fe-PDTA......S34**
16. **References......S35**

1. General experimental considerations

1.1. Materials and reagents: All chemicals and solvents were purchased from commercially available sources (Sigma-Aldrich, Oakwood Chemical, and ThermoFisher Scientific) and were used without any further purification. Super conductive carbon black (C65), polyvinylidene fluoride (PVDF), carboxymethyl cellulose (CMC), styrene-butadiene rubber (SBR), and graphite powder was purchased from Xiamen Tmax Battery Equipments Limited. Carbon paper (Sigracet 39 AA) was purchased from Fuel Cell Store and used without any further treatment. Ultrapure deionized (DI) water (18.2 MΩ·cm, Thermo-Scientific) was used for all solution preparations and experiments.

1.2. Synthesis of Li[Fe-PDTA]:[1] In a 1000 mL bottle with a stir bar, 400 mL of 0.75 M LiOH solution was prepared and heated to 70 °C. 1,3-Diaminopropane-*N*,*N*,*N*′,*N*′-tetraacetic acid (1,3-PDTA, 30.63 g, 100 mmol) was added to the LiOH solution, and the mixture was stirred at 90 °C for 1 hour. $FeCl_3\cdot6H_2O$ (24.33 g, 90 mmol) was dissolved in 200 mL of DI water in a 250 mL beaker. The $FeCl_3$ solution was then added dropwise to the mixture using a Pasteur pipette. The reaction was stirred overnight at 70 °C. Afterward, insoluble solid was removed by vacuum filtration and washed with 100 mL of DI water. The filtrate was gently heated to evaporate water, reducing the volume to approximately 150 mL and yielding a dark yellow solution. After cooling the filtrate to room temperature, excess isopropyl alcohol (IPA, ~400 mL) was added to the filtrate to precipitate a yellow solid. The solid was collected by vacuum filtration to remove free ions and washed with ice-cold water (50 mL) followed by IPA (2 × 100 mL). The product was dried overnight in an oven at 70 °C, yielding a slightly greenish-yellow powder (19.6 g, 52.0%). HRMS (ESI): Exact mass calculated for $[M]^-$, $[C_{11}H_{14}FeN_2O_8]^-$ = 358.0099, found *m*/*z* = 358.0103, mass error = 1.1 ppm. TGA: 8.94% weight loss of Fe-PDTA at 150 °C under N_2, indicating dihydrate form. ICP: Li/Fe molar ratio = 0.97. FTIR (neat, cm^{-1}): 3473, 3284, 2965, 2935, 2161, 2021, 1973, 1627, 1471, 1438, 1427, 1360, 1345, 1309, 1233, 1154, 1095, 1064, 1050, 1036, 1013, 1000, 975, 961, 934, 918, 877, 846, 822, 810, 738, 715, 689, 624, 616.

1.3. Cyclic voltammetry (CV) measurements: CV measurements were performed using BioLogic BP-300 potentiostat with a three-electrode cell configuration, consisting of a glassy carbon (GC) electrode (3 mm diameter), an Ag/AgCl (saturated KCl solution) reference electrode, and a Pt wire counter electrode. The experiments were conducted in a borate buffer electrolyte (pH 9.0) at a scan rate of 1.0 mV/s. A degraded $LiFePO_4$ (D-LFP) working electrode was prepared by coating a slurry of D-LFP onto the GC electrode and drying it in a vacuum at 70 °C. The slurry contained D-LFP, carbon black (Super C65), and polyvinylidene fluoride (PVDF) in N-methyl pyrrolidone (NMP) solvent with a weight ratio of D-LFP:C65:PVDF = 8:1:1. The Fe-PDTA concentration in the CV measurement was 10 mM.

1.4. Powder X-ray diffraction (PXRD): PXRD measurements were performed on a Bruker D8 Advance X-ray diffractometer with Cu K*α* radiation (λ = 1.54056 and 1.54439 Å, 1:0.5 ratio) at room temperature. PXRD patterns were measured between the range of 15–50° with a step size of 0.01° and an exposure time of 1.5 seconds per step. The measured patterns were matched to $LiFePO_4$ (ICSD-15448) and $FePO_4$ (ICSD-92199) patterns from the ICSD database. PXRD measurements for Rietveld refinement were recorded between the range of 10–80° with a step size of 0.01° and an exposure time of 6 seconds per step. Rietveld refinement was done using the FullProf

software.[2] For Rietveld refinement, the initial atomic coordinates and cell parameters for $LiFePO_4$ and $FePO_4$ were taken from Andersson et al.[3], assuming a Li–Fe antisite defect level of 2%. Background, sample displacement, peak shape, asymmetry parameters, atomic coordinates, cell parameters, and Li and Fe site occupancies were refined for each sample independently. The background was fitted using a linear interpolation method, and peak shape was refined using a pseudo-Voigt function. Preferred orientation correction was applied after refinement of the atomic coordinates and cell parameters. For Li and Fe site occupancy refinement, constraints were applied to ensure that the sum of Li and Fe occupancies at both the Li and Fe sites equals 0.5 (multiplicity factor of 4 for both sites).

1.5. Inductively coupled plasma optical emission spectrometry (ICP-OES): ICP-OES measurements were conducted using an Agilent 5800 ICP-OES. 10 mg of $LiFePO_4$ (LFP) samples were dissolved in a mixture of 1 mL of nitric acid and 3 mL of hydrochloric acid in a 15 mL conical tube. The mixture was heated at 60 °C for 2 hours and then left to rest overnight at room temperature. The digested solutions were diluted with 2% nitric acid to a final concentration of 10 mg/L. Before analysis, the solution was filtered using a hydrophilic filter (0.22 μm, EZFlow HP syringe filter, 37B-2116-OEM) to remove carbon.

1.6. Fourier-transform infrared (FTIR) spectroscopy: FTIR spectra were obtained by using diamond crystal attenuated total reflection (ATR) geometry on a Bruker Tensor 27 spectrometer at room temperature. Scan range was 4000–600 cm^{-1} with resolution of 2 cm^{-1} and accumulations of 64 scans.

1.7. High-resolution transmission electron microscopy (HR-TEM): HR-TEM images were obtained using an FEI Tecnai TF-30 operated at 300 kV. For TEM specimen preparation, LFP samples were dispersed in acetone at a concentration of 0.1 mg/mL. TEM specimens were prepared by dropping 20 μL of the dispersion onto the shiny side of Au TEM grids with a lacey carbon support film (LC400-AU-150). The specimens were then dried at 70 °C overnight. Before analysis, plasma cleaning of TEM specimens was performed for 15 min using Ar/H_2 (5%) gas.

1.8. Solution-based post-regeneration treatment: Solution-based post-regeneration treatment for regenerated $LiFePO_4$ (R-LFP) was performed as follows: A 20 mL chelating solution of 1 M *N*-(2-hydroxyethyl)ethylenediamine-*N*,*N′*,*N′*-triacetic acid (HEDTA) was prepared, and its pH was adjusted to 5.5 using LiOH. Then, 2.0 g of R-LFP and a stir bar were added to a 22 mL vial. The 1.0 M HEDTA (pH 5.5) solution was added to the vial containing R-LFP, and the mixture was stirred at 1500 rpm for 24 h at room temperature. After the reaction, the stir bar was removed, and the suspension was collected by centrifugation (Savant™ SPD131DDA SpeedVac) at 1725 rpm for 10 min. The remaining HEDTA solution was retained for potential reuse. The resulting solid was stirred in DI water for 15 min, collected by vacuum filtration, washed sequentially with DI water and acetone, and dried in an oven at 70 °C. The final product, referred to as RT-LFP, was 1.50 g (75 wt.%).

1.9. X-ray photoelectron spectroscopy (XPS): XPS spectra were measured using a Thermo Scientific K-alpha XPS equipped with a monochromatic Al K*α* X-ray source and spot size of 400 μm. The LFP samples were pressed onto the sample holder and mounted on a sample stage placed in the analysis chamber. The X-ray gun was operated at 15 kV and 20 mA. An electron flood gun was used during measurements. Survey scans were collected from 10 to 1300 eV with a step size of 1 eV. The binding energy of the C 1s peak at 284.5 eV was used as a charge reference in the XPS spectra. High-resolution scans for Fe 2*p* were acquired from 700 to 740 eV with a step size

of 0.1 eV. Curve-fitting was performed using CasaXPS software with a mixture of Gaussian (70%) and Lorentzian (30%) functions and a Shirley background.[4] For fitting the Fe 2*p* spectra, the spectrum of P-LFP was first fitted under the assumption that no Fe^{III} was present, to extract the Fe^{II} peak parameters by constraining spin-orbit split peak ratios. In P-LFP, the initial guesses for Fe $2p_{3/2}$ peaks were 709.5 and 710 eV, and for Fe $2p_{1/2}$ peaks, 723 and 724 eV.[5,6] Based on the obtained relative binding energies, full width at half maximum (FWHM), and areas of the Fe^{II} components, the Fe 2*p* spectrum of D-LFP was then fitted to resolve both Fe^{II} and Fe^{III} contributions. Finally, the Fe 2*p* spectra of R-LFP and RT-LFP were fitted using the Fe^{II} and Fe^{III} peak parameters determined from P-LFP and D-LFP.

1.10. ^{57}Fe Mössbauer spectroscopy: ^{57}Fe Mössbauer spectroscopy were performed using a 1024 channel See Co model W304 resonant gamma-ray spectrometer using ^{57}Co on Rh foil as a gamma-ray source. The isomer shift and velocity scale were calibrated with a *α*-Fe foil as the reference absorber at room temperature (295 K). LFP samples (30 mg) were loaded into the sample chamber with a 15 mm diameter to achieve a natural iron mass loading of approximately 6 mg/cm^2. Spectra were collected under vacuum with a source velocity range of ±6 mm/s. Spectra were fitted to appropriate combinations of Lorentzian doublets using VindaD Excel add-in.[7]

1.11. Electrochemical performance measurements: The electrodes were prepared by a typical slurry-coating method. The LFP slurry was prepared by mixing 80 wt.% LFP powder, 10 wt.% C65, and 10 wt.% PVDF in NMP solvent. The slurry was uniformly coated onto aluminum foil and dried at 70 °C for 12 h. The dried LFP electrode was then punched into 13 mm diameter disks. The areal mass loading of the LFP active material was 3.0–5.0 mg/cm^2. The graphite slurry was prepared by mixing 93 wt.% graphite powder, 2.5 wt.% CMC, 2.5 wt.% SBR, and 2 wt.% C65 in DI water. The resulting slurry was cast onto copper foil and dried at 70 °C for 12 h. The graphite electrode was punched into 18 mm diameter disks. Electrochemical performance of the electrodes was evaluated by assembling coin cells. CR2032 coin cells were assembled in an argon-filled glovebox using Celgard 2320 membrane as a separator. A fixed amount of 60 µL electrolyte, 1 M $LiPF_6$ in a 1:1:1 (*v*/*v*/*v*) mixture of ethylene carbonate (EC), dimethyl carbonate (DMC), and diethyl carbonate (DEC) with 5 wt.% fluoroethylene carbonate (FEC) additive, was used. Electrochemical measurements were conducted using a Neware (WHW-25L-S-16CH) battery cycler. Half-coin cells were assembled using the LFP electrode as the working electrode and lithium metal foil as the counter/reference electrode. Full-coin cells were assembled with an LFP electrode as the cathode and a graphite electrode as the anode, with the capacity ratio between the negative and positive electrode (N/P ratio) between 1.1 and 1.2. Before electrochemical evaluation, three formation cycles were performed at 0.1 C-rate (1 C-rate for LFP: 170 mA/g) within a potential range of 2.5–3.8 V (*vs*. Li/Li^+) to form stable solid electrolyte interphase layers, followed by cycling at higher rates.

Single-layer full-pouch cells were assembled using an LFP cathode (24.08 cm^2), a graphite anode (26.10 cm^2), and Celgard 2320 separator in an argon-filled glovebox. A fixed amount of 200 µL of 1 M $LiPF_6$ (1:1:1, *v*/*v*/*v*, EC/DMC/DEC with 5 wt.% FEC) was used. The cathode and anode were matched to achieve an N/P ratio of 1.0–1.3. The mass loading of P-LFP and RT-LFP in **Figure 5e** was 3.67 mg/cm^2 and 4.96 mg/cm^2, respectively. After assembly, a tap charge was applied by charging the cells to 1.5 V at the rate of 0.1 C for 2 h, followed by a 12 h rest to allow for electrolyte wetting. Prior to long-term cycling, three formation cycles were performed between 2.5 V and 3.8 V at 0.1 C, followed by a constant-voltage hold unit the current density decreased to 0.05 C. Initial

capacities were measured using three cycles at C/3, after which extended cycling tests were conducted at 1 C, with three C/3 cycles inserted every 50 cycles to quantify capacity fade within the same voltage range, followed by a constant-voltage hold until the current density decreased to 0.2 C. The specific capacity was calculated using only the constant-current discharge portion.

1.12. High-resolution mass spectrometry (HRMS): HRMS result of a compound was obtained using a Thermo Q ExactiveTM Plus in the mass spectrometry facility at the University of Wisconsin Madison. Data were collected by the facility staff.

1.13. Thermogravimetric analyzer (TGA): TGA measurements were conducted on the TA Instruments Q50 with a heat rate of 10 °C/min from room temperature to 800 °C under N_2 flow (50 mL/min).

2. Isolation of degraded $LiFePO_4$ from spent battery packs

Commercial spent LFP battery packs (K2 Energy K2B24V10EB 24 V 9600 mAh) were purchased from eBay. After discharging, the battery packs were disassembled into individual 26650 LFP cells (**Caution!** Avoid short circuits during disassembly). The capacity of each 26650 LFP cells (K2 Energy LFP26650EV 3.2V 3200 mAh) was measured with the voltage range of 2.0–4.0 V at 1.6 A using a BioLogic BP-300 potentiostat and HCV-3048 booster. 26650 LFP cells with 45–60% of their original capacity were selected.

a Disassembly of a spent $LiFePO_4$ battery pack

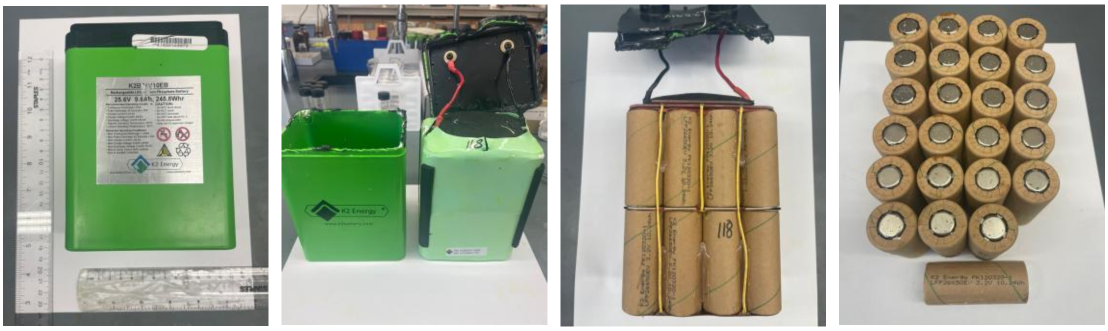

b Voltage profiles of spent $LiFePO_4$ cells at 1.6 A

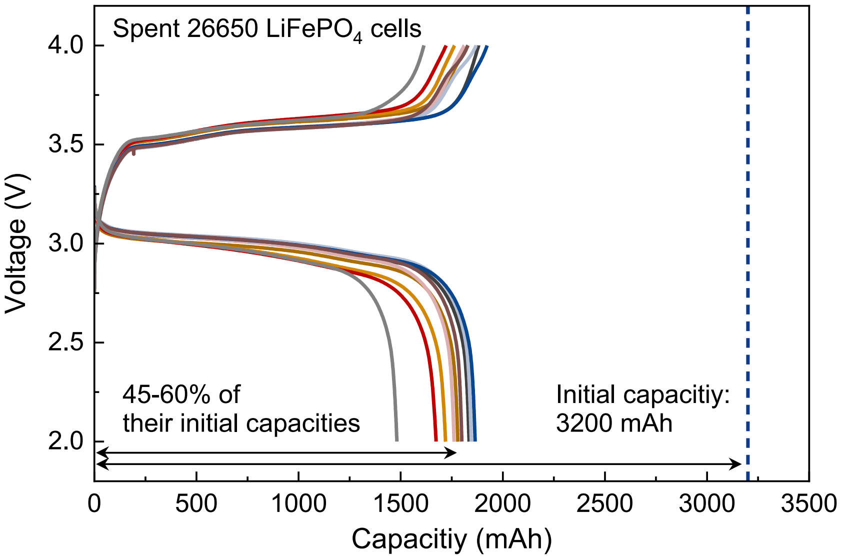

Figure S1. (a) Disassembly of a spent LFP battery pack. (b) Charging-discharging voltage profiles of selected spent 286650 LFP cells at 1.6 A within a voltage range of 2.0 to 4.0 V, remaining 45–60% capacity.

To ensure safety, the LFP cells were fully discharged in saturated NaCl solution overnight before disassembly. After drying, the cells were manually disassembled into cathodes (Al foil), anodes (Cu foil), separators, and shells. The cathode strips were soaked in DI water and sonicated for 0.5–1.0 h to peel off the cathode film from the Al foil. The detached cathode films were washed with DI water and dried overnight in a 70 °C oven. The dried cathode films were ground into powders and stirred in NMP overnight at 80 °C to dissolve the binder. The solids were collected from the suspension containing binder and carbon by centrifuging (Beckman Avanti™ J-25 and JLA-10.500) at 4000 rpm for 5 min. The precipitated solids were re-dispersed in NMP, and this process was repeated three times to remove more of the binder. Remaining carbon and binder impurities were further removed by dispersing the precipitated powders in DI water. The mixture was stirred overnight and allowed to settle for phase separation. D-LFP powders settled at the bottom, while impurities including carbon and binder floated at the top. These impurities were removed by decantation. The D-LFP powders were re-dispersed in DI water, and this process was repeated five times. The obtained D-LFP powders were washed with acetone and dried for regeneration.

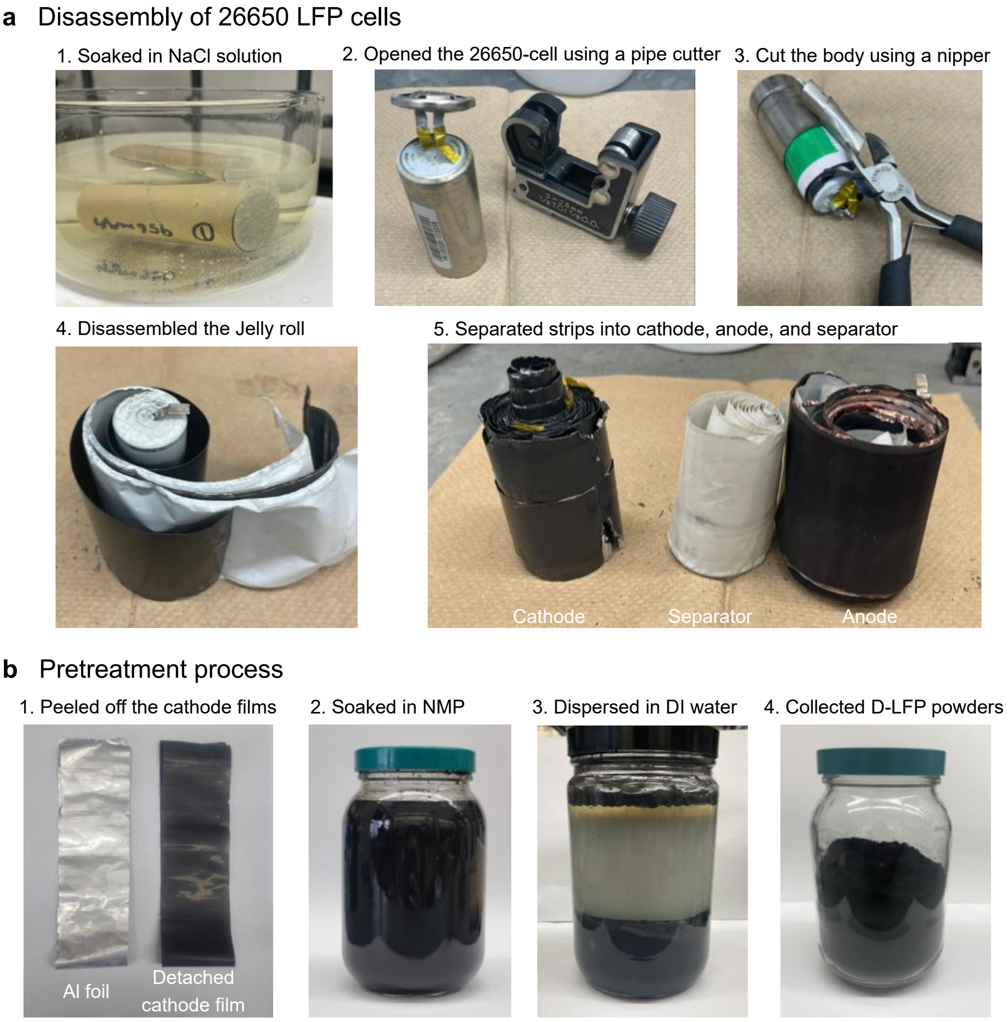

Figure S2. (a) Disassembly process for 26650 LFP cells. (b) Pretreatment process for collecting D-LFP powders from the spent cathode strips.

3. Kinetics of the reactions between degraded $LiFePO_4$ and three different mediators

Kinetics of the reactions between D-LFP and three different mediators, Fe^{II}-PDTA, Fe^{II}-EDTA, and anthrahydroquinone-2,7-disulfonate (AHQDS), were tested in batch experiments with excess reduced mediators. A series of parallel reactions were performed as follows. First, 20 mL of 0.3 M reduced mediator solution in 1 M lithium borate was prepared by electrochemical reduction in a flow cell. Then, 0.5 g of D-LFP and a 2 cm star-shaped stir bar were placed in a 24 mL vial. The reduced mediator was added to the vial using a syringe and allowed to reduce D-LFP for a specified time under an N_2 atmosphere. After the reaction, the solid was rapidly collected by vacuum filtration, washed with excess DI water and acetone, and then dried in an oven at 70 °C for 3 h. The recovered solid materials were analyzed by PXRD, and the LFP fraction was quantified using Rietveld refinement (see Section 1.4 in General Considerations) excluding Li–Fe antisite defects.

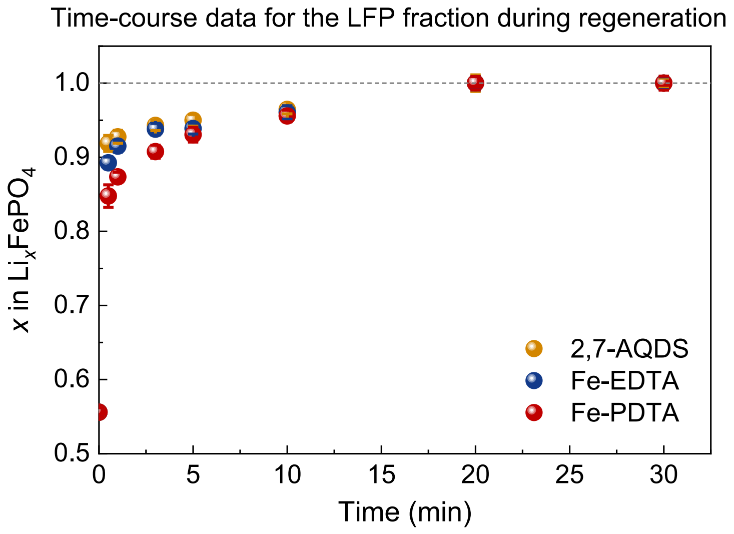

Figure S3. Time-course data for the LFP fraction in D-LFP during regeneration under excess reduced mediator conditions (0.5 g of D-LFP and 20 mL of 0.3 M reduced mediator). The fractions were quantified from the powder X-ray diffraction data in **Figure S4** using Rietveld refinement. Error bars correspond to the statistical uncertainties obtained from the Rietveld refinement. The D-LFP used in these experiments was isolated from spent LFP cells with similar remaining capacities (40–55%), but it belongs to a different batch.

Time-course PXRD results of LFP samples during regeneration under excess reduced mediators

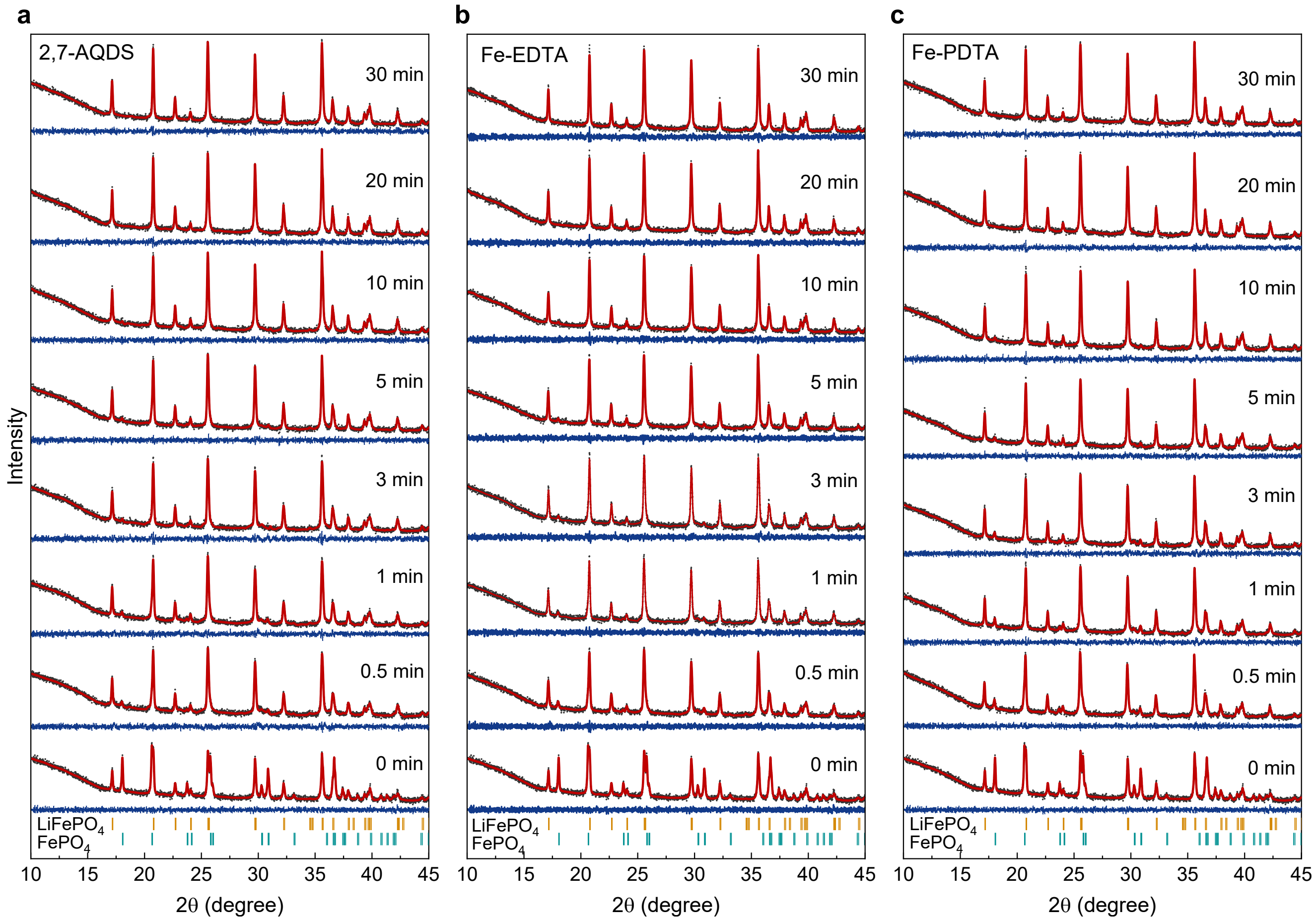

Figure S4. Time-course powder X-ray diffraction (PXRD) results of LFP samples during regeneration under excess reduced mediators. (a) 2,7-AQDS, (b) Fe-EDTA, and (c) Fe-PDTA. For each time point, 0.5 g of D-LFP was reacted with 20 mL of 0.3 M reduced mediator under 1000 rpm stirring. Black dots and red lines represent the experimental data and the calculated Rietveld refinement fits, respectively, and the blue lines indicate the difference between the experimental and fitted curves.

4. Small-scale regeneration of $LiFePO_4$ in an H-cell

Small-scale regeneration reaction was conducted in H-cell with continuous electrolyte flow system, as shown in **Figures 2c** and **S5**. This electrochemical cell consisted of an empty chromatograph cartridge (Biotage® Sfär Silica HC D 10g, FSUD-0443-0010) as a catholyte reservoir to contain D-LFP and two compartments (cathodic and anodic chambers) separated by a cation-exchanged membrane of Nafion 115. A 3.0×2.5 cm piece of Nafion 115 was cut and soaked in 1.0 M Li_2SO_4 solution overnight before assembly the cell. The H-cell was assembled around the Nafion 115 using a Viton O-ring and clamp to secure the cell. Each chamber and the catholyte reservoir were equipped with a stir bar. To the catholyte reservoir was added 300 mg of D-LFP and sealed with a 14/20 rubber septum. To the cathodic chamber was added 60 mg of Fe-PDTA (0.15 mmol) and 15 mL of the borate buffer electrolyte (pH 9.0). A 12 mm diameter piece of carbon paper was used as a working electrode and was suspended using a glassy carbon electrode holder. A 14/20 rubber septum was punctured with a 14-gauge needle. Then, the working electrode, Ag/AgCl reference electrode, and polytetrafluoroethylene (PTFE) tube (OD 2 mm) were threaded through the rubber septum before removal of the needle, respectively. The electrodes were then placed in the cathodic chamber roughly 5 mm above the stir bar and the 14/20 joint sealed with the rubber septum. To the anodic chamber, 10 mL of the borate buffer electrolyte was added. A Pt-wire (ca. 4 cm, 5–7 spiral coils) was affixed to copper wire as a counter electrode. A 14/20 rubber septum was punctured with a 14-gauge needle, and the copper wire (with Pt counter electrode) and PTFE tube (OD 2 mm) were threaded through the rubber septum before removal of the needle. The Pt counter electrode was then placed in the anodic chamber roughly 5 mm above the stir bar and the 14/20 joint sealed with the rubber septum.

The cathodic chamber and catholyte reservoir were connected by two Pharmed® BPT pump tubes (MasterFlex, VA-06508-14) for catholyte circulation. For connection of the cathodic chamber to the catholyte reservoir, a pump tube was affixed to 16-gauge needle (length: 150 mm) and 18-gauge needle (length: 25 mm) using male Luer adapters (VWR, MFLX30800-24). The 16-gauge needle was inserted into the cathodic chamber at the same height as the anolyte, while the 18-gauge needle was inserted into the catholyte reservoir. For connection of the catholyte reservoir to the cathodic chamber, a pump tube was affixed to female Luer adaptor (VWR, MFLX30800-08) and 16-gauge needle (150 mm) with male Luer adapter (VWR, MFLX30800-24). The female Luer adaptor was connected to the bottom of catholyte reservoir, and the 16-gauge needle was inserted into the catholyte reservoir roughly 5 mm above the stir bar. A peristaltic pump (Cole Parmer Masterflex L/S) circulated around 5 mL of catholyte at a flow rate of 10 mL/min. Catholyte and anolyte solutions were bubbled with N_2 gas through PTFE tubes for 30 min while circulating the catholyte. After bubbling, the PTFE tubes were positioned above the solutions. Both catholyte and anolyte were stirred at 1000 rpm.

Electrochemical regeneration reactions were performed using a BioLogic BP-300 potentiostat in constant-current electrolysis mode at −1 mA/cm^2 until the potential reached −0.8 V vs Ag/AgCl with continued catholyte circulation and N_2 flow. On completion of the reaction, regenerated LFP (R-LFP) was collected by vacuum filtration, washed with DI water and acetone to remove electrolyte and redox mediator, and then dried at 70 °C. The final mass of R-LFP was 282 mg (94.0 wt.%).

a Setup for the small-scale regeneration reaction in an H-cell

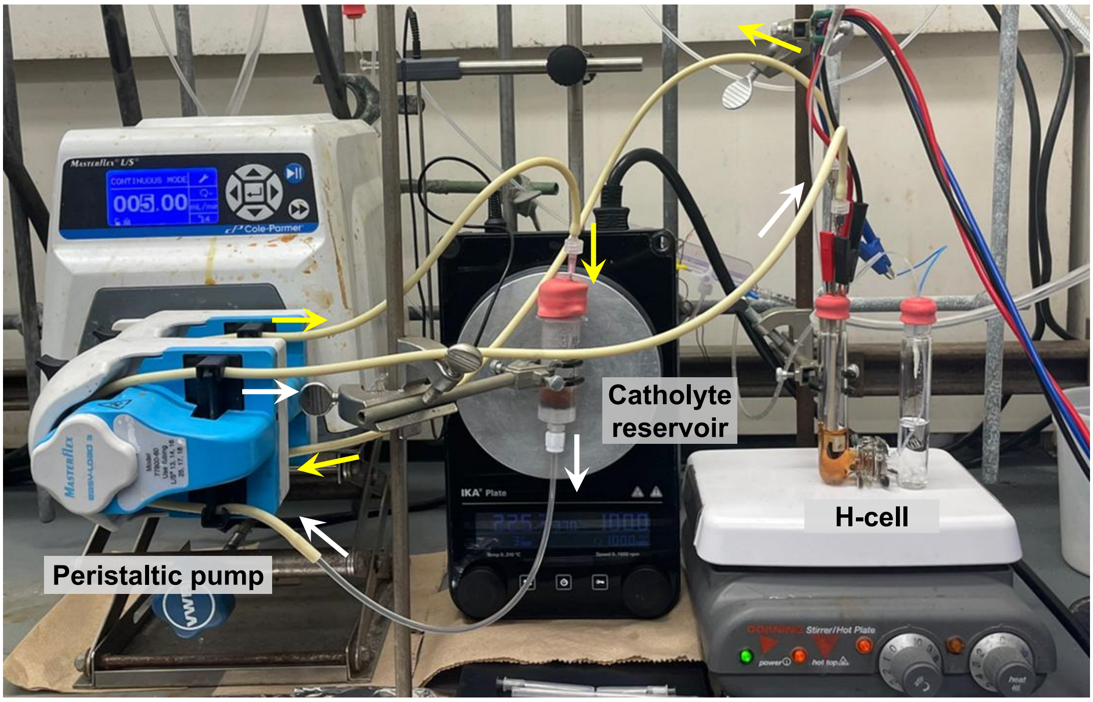

b H-cell configuration before assembly

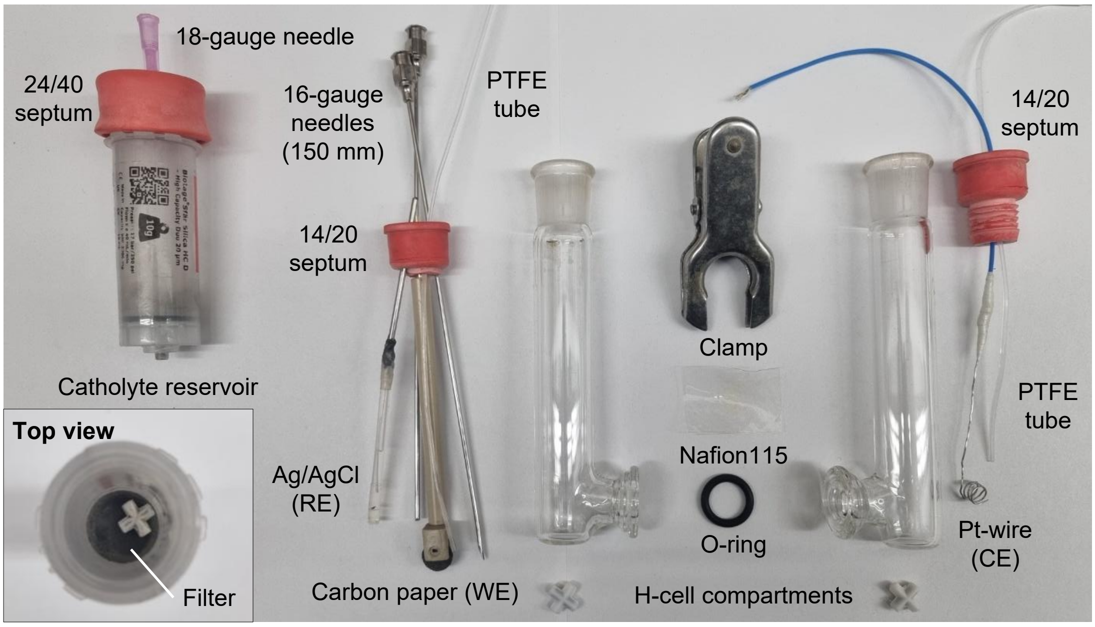

Figure S5. (a) Setup for the small-scale regeneration reaction in an H-cell. Yellow arrows indicate the flow of Fe-PDTA solution from the flow cell to the catholyte reservoir, and vice versa for the white arrows. (b) H-cell configuration before assembly. Inset image: Top view of the catholyte reservoir.

5. Evaluation of Faradaic efficiency

The theoretical charge required for D-LFP regeneration was determined in a chemical reduction experiment in which the amount of Fe^{II}-PDTA needed to achieve full reduction/relithiation of D-LFP was quantified by UV-vis spectroscopy. First, 23 mL of 0.1 M Fe^{II}-PDTA solution was prepared by electrochemical reduction in a flow cell. Then, 300 mg of D-LFP was reacted with 20 mL of 0.1 M Fe^{II}-PDTA for 1 h under stirring at 1000 rpm in an N_2-filled purge box. After completion of the reaction, a 1 mL aliquot was filtered through a 22 µm syringe filter to remove R-LFP. The filtrate was sequentially diluted by a factor of 500 with N_2-saturated 1 M borate buffer to prepare 0.2 mM Fe-PDTA solution. The diluted solutions collected before and after the reaction were transferred to a sealed quartz cuvette under N_2 and analyzed by UV-vis absorption spectroscopy. The Faradaic efficiency was calculated by the following equations:

$$\text{Faradaic efficiency (\%)} = \frac{z \times n \times F}{Q} \times 100 \qquad \text{(eq 1)}$$

$$n\ \text{(mol)} = C_{\text{Fe(III)}} \times V \qquad \text{(eq 2)}$$

$$A = (\varepsilon_{\text{Fe(III)}} C_{\text{Fe(III)}} + \varepsilon_{\text{Fe(II)}} (C_{\text{Total}} - C_{\text{Fe(III)}})) \times l \qquad \text{(eq 3)}$$

$$C_{\text{Total}} = C_{\text{Fe(III)}} + C_{\text{Fe(II)}} \qquad \text{(eq 4)}$$

where Q is the net charge passed for regeneration of D-LFP during the reaction, z is the number of electrons, n is the number of moles of Fe^{II}-PDTA consumed, F is the Faraday constant (96,485 C/mol), A is the absorbance of Fe-PDTA solution after the reaction, ε is the molar extinction coefficient, C is the Fe-PDTA concentration (M), l is the optical path length (1 cm), V is the volume of Fe-PDTA solution. The concentrations in 0.2 mM $Fe^{II/III}$-PDTA mixture after the reaction were determined by linear combination fitting of UV-vis absorption spectra of Fe^{II}-PDTA and Fe^{III}-PDTA (**Figure S6** and eq 3). This analysis yields a near-unity Faradaic efficiency.

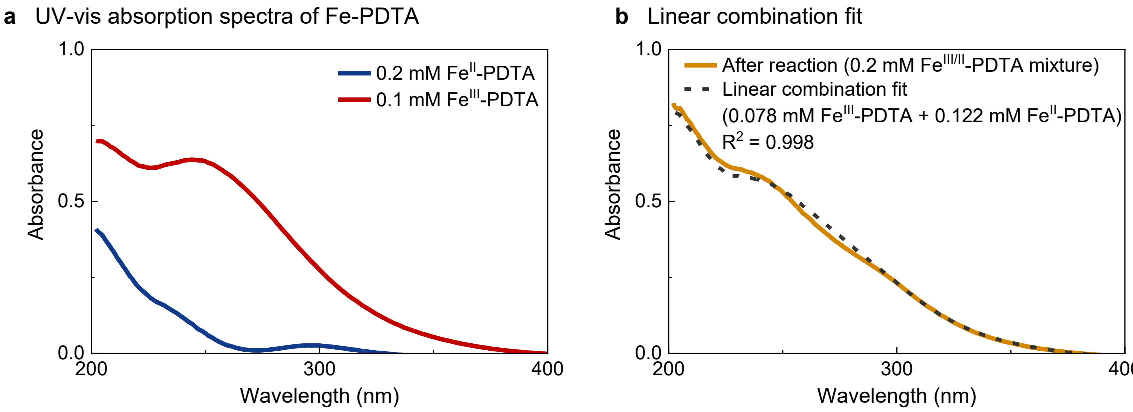

Figure S6. UV-vis absorption spectra of Fe-PDTA. (a) 0.2 mM Fe^{II}-PDTA and 0.1 mM Fe^{III}-PDTA in 1 M borate. (b) 0.2 mM $Fe^{II/III}$-PDTA mixture after reaction in 1 M borate and linear combination fit using the UV-vis absorption spectra of Fe^{III}-PDTA and Fe^{II}-PDTA in panel (a).

6. Large-scale regeneration of $LiFePO_4$ in a flow cell

The setup for 10 g and 100 g-scale regeneration reaction is shown in **Figures S8** and **S9**. No special effort was applied to maintain anaerobic conditions in the large-scale experiments. For the large-scale reaction, a catholyte containing 0.3 M Fe-PDTA in 1.0 M boric acid solution (pH 6.7) was used to prevent insoluble precipitation caused by the high concentration of Fe-PDTA during the reaction. The anolyte was the same as that used in the small-scale reaction (1.0 M borate buffer, pH 9.0). A membrane-electrode assembly supplied from Fuel Cell Technologies Inc. was used as the flow apparatus. The flow cell consisted of a Nafion 117 membrane, a cathode stack of four carbon papers (4 cm^2), and a commercial nickel foam anode (4 cm^2). The cell was enclosed by PTFE gaskets, graphite blocks with interdigitated flow channels (channel width = 0.81 mm, depth = 1.0 mm, lands between channels = 0.76 mm), and gold-plated copper current collectors. An empty chromatograph cartridge (Biotage® Sfär Silica C18 D 60g, FSUD-0401-0060) served as the catholyte reservoir, and a 500 mL round-bottom flask (RBF) was used as the anolyte reservoir. Catholyte and anolyte were circulated through the flow cell compartments with a peristaltic pump (Cole Parmer Masterflex L/S) and Pharmed® BPT pump tubing (MasterFlex, VA-06508-14). The catholyte reservoir contained 70 mL of 0.3 M Fe-PDTA solution with a stir bar, while the anolyte reservoir contained 210 mL of borate electrolyte.

Electrochemical regeneration reactions were performed with a BioLogic BP-300 potentiostat and HCV-3048 booster in constant-current electrolysis mode at −100 mA/cm^2 with fluid circulated at 50 mL/min until the cell voltage reached −2.8 V. All reactions were conducted under atmospheric conditions, with the stir bar in the catholyte reservoir set to 300 rpm. Before the regeneration reactions, Fe^{III}-PDTA was pre-reduced to Fe^{II}-PDTA without D-LFP. After Fe-PDTA reduction, LiOH in the anolyte was replenished based on the charge passed (Q = 1674 C, equivalent to 728 mg of $LiOH{\cdot}H_2O$). Then, 10 g of D-LFP was added to the catholyte reservoir, and the solution was circulated for 3 min. Electrochemical regeneration was then carried out under the same conditions. After the reaction, Fe^{II}-PDTA solution was transferred to a volumetric flask, with approximately 0.5 mL lost during collection. R-LFP in the catholyte reservoir was collected by vacuum filtration and washed with DI water and acetone. Additional LiOH was added to the anolyte depending on the charge passed (Q = 1944 C equivalent to 845 mg of $LiOH{\cdot}H_2O$). Fe^{II}-PDTA solution and 10 g of D-LFP were added to the catholyte reservoir, and the solution was circulated for 3 min before subsequent reactions. Electrochemical regeneration was repeated three times to demonstrate a closed-loop recycling system. The average mass of R-LFP from three experiments was 9.62 ± 0.06 g (96.2 wt.%).

For the 100 g scale reaction, a large catholyte reservoir (Biotage® Sfär DLV Empty 350g, DLV-0000-0350) was employed along with 200 mL of 0.3 M Fe-PDTA catholyte and 2000 mL of anolyte, while the rest of the setup was identical to that used in the 10 g scale reaction. The electrochemical reaction was performed in constant-current electrolysis mode at −50 mA/cm^2 with fluid circulated at 30 mL/min until the cell voltage reached −2.4 V. The reduced current density was applied to accommodate the increased flow resistance resulting from the large quantity of D-LFP. The mass of R-LFP was 98.1 g (98.1 wt.%).

Catholyte reservoir size depending on the reaction scale

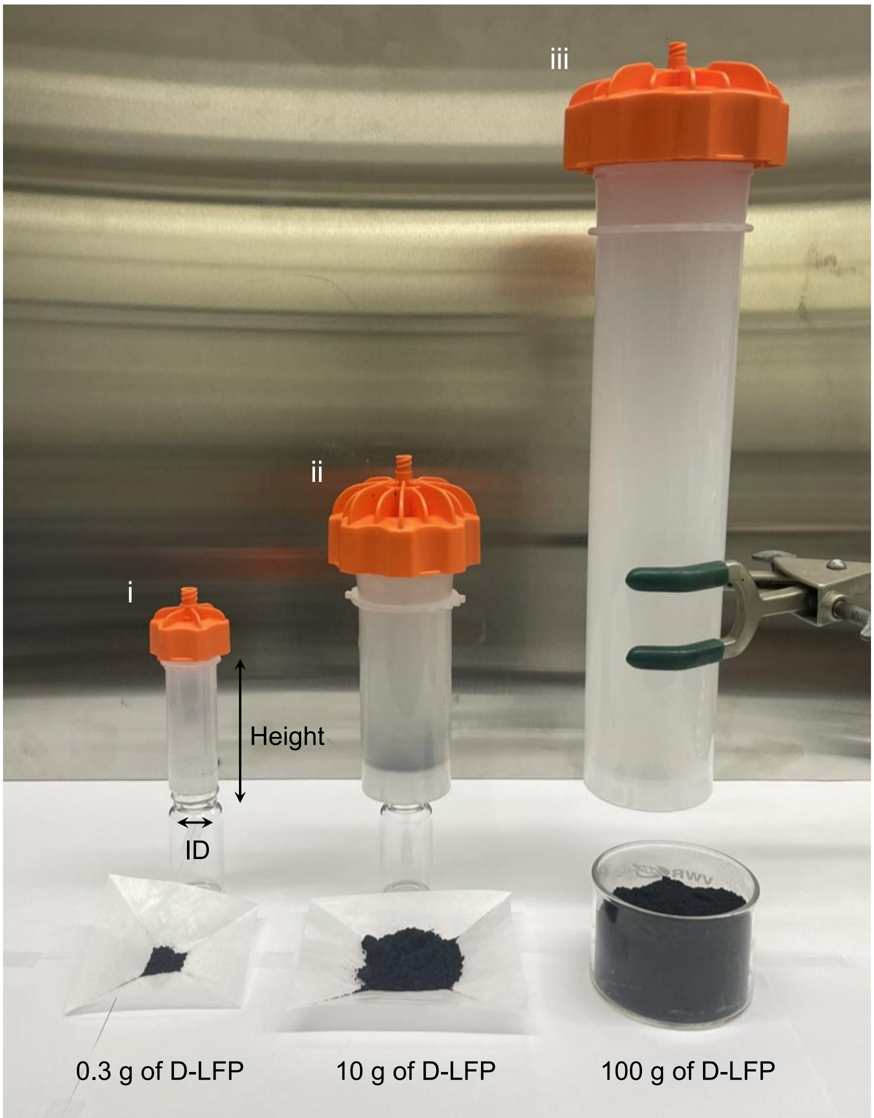

Figure S7. Different catholyte reservoir sizes depending on the reaction scale. (i) small scale reaction reservoir (ID = 21.1 mm, height = 72 mm), (ii) 10 g-scale reaction reservoir (ID = 38.7 mm, height = 110 mm), and (iii) 100 g-scale reaction reservoir (ID: 61.3 mm, height: 300 mm).

a Setup for a 10 g-scale regeneration reaction in a flow cell

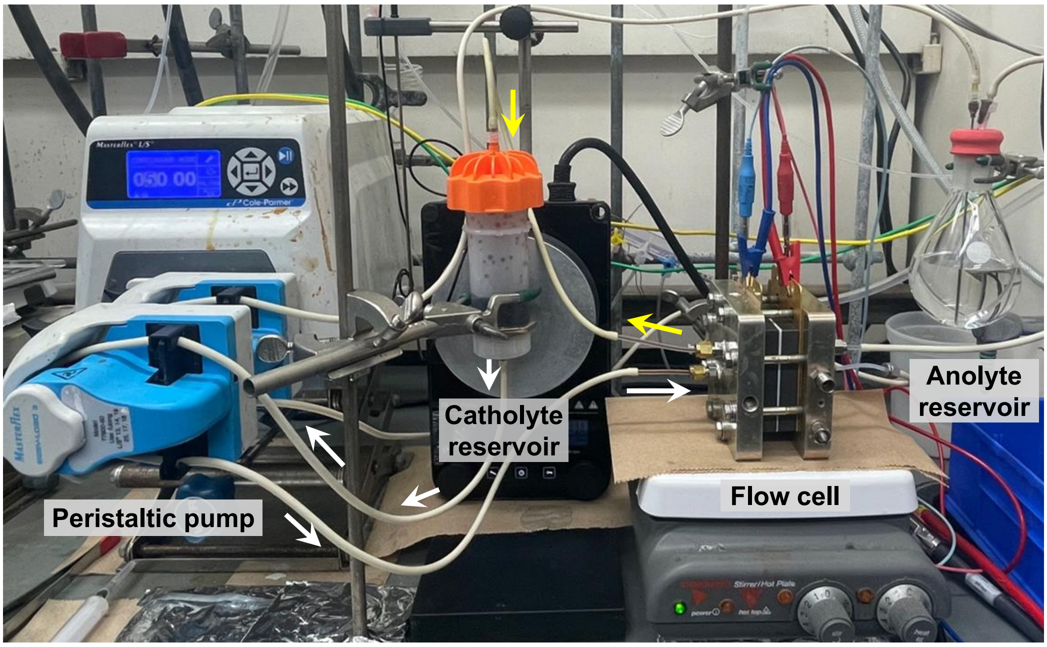

b Flow cell configuration before assembly

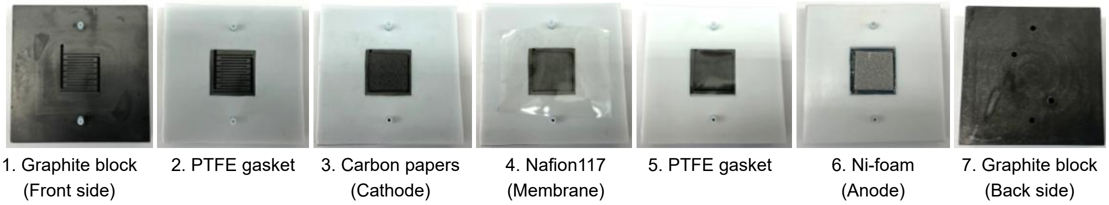

c Pictures of the catholyte reservoir

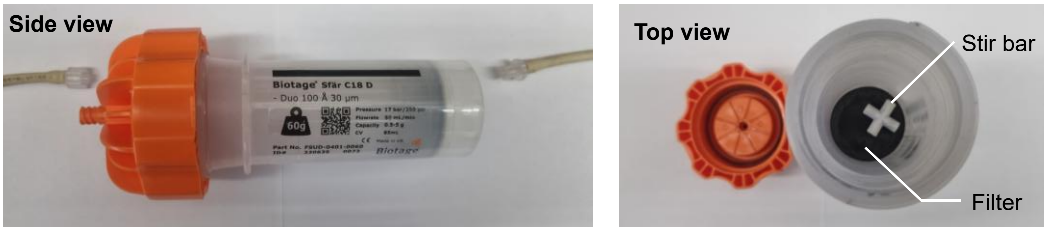

Figure S8. (a) Setup for the large-scale regeneration reaction in a flow cell. Yellow arrows indicate the flow of Fe-PDTA solution from the flow cell to the catholyte reservoir, and vice versa for the white arrows. (b) Flow cell configuration before assembly. (c) Side and top views of the catholyte reservoir.

Setup for a 100 g-scale regeneration reaction in a flow cell

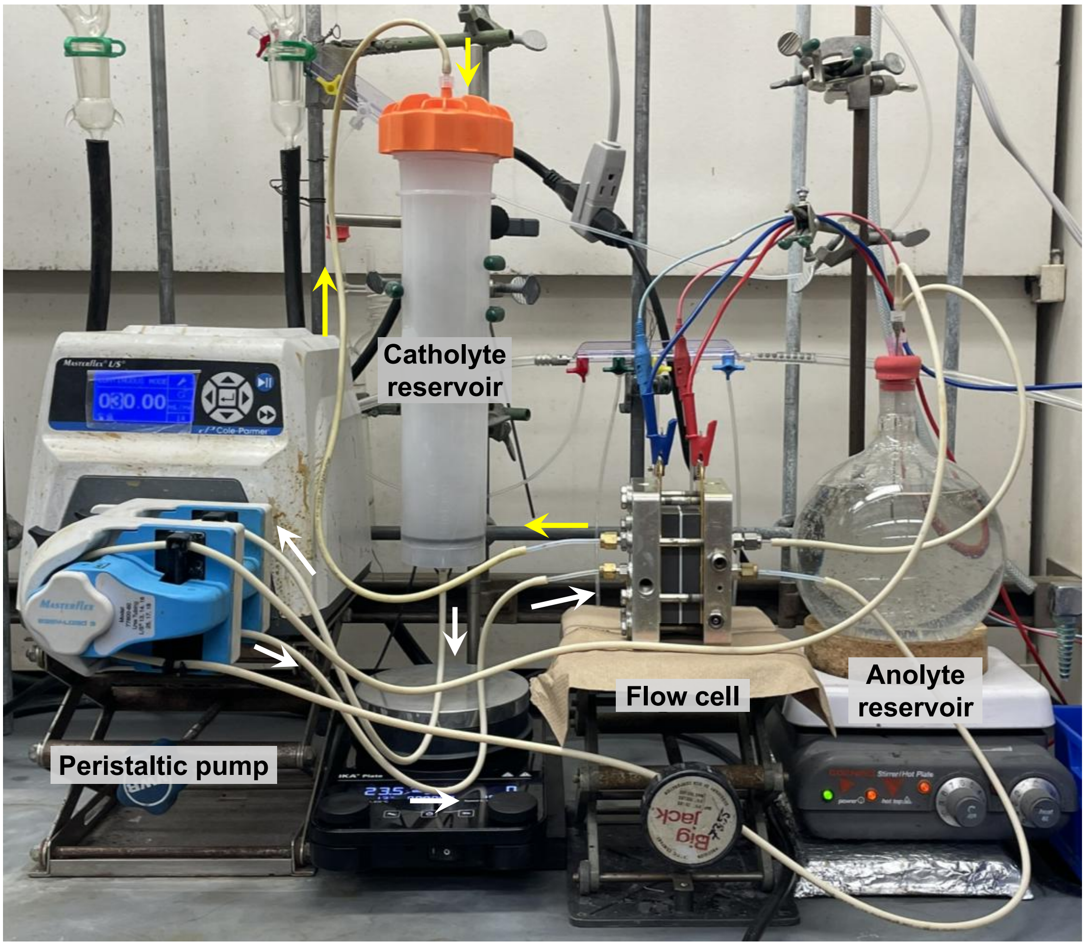

Figure S9. Setup for a 100 g-scale regeneration reaction in a flow cell.

LSV curves of the flow cell

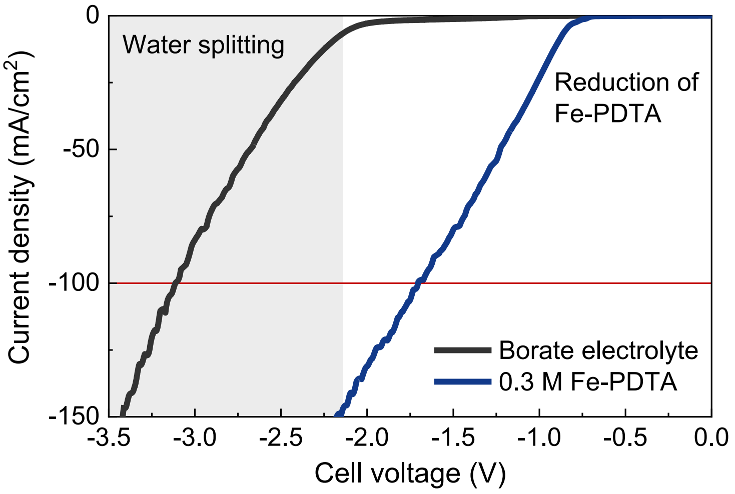

Figure S10. Linear-sweep voltammetry (LSV) curves of bare electrolyte and 0.3 M Fe-PDTA in the flow cell at a scan rate of 10 mV/s and a flow rate of 50 mL/min.

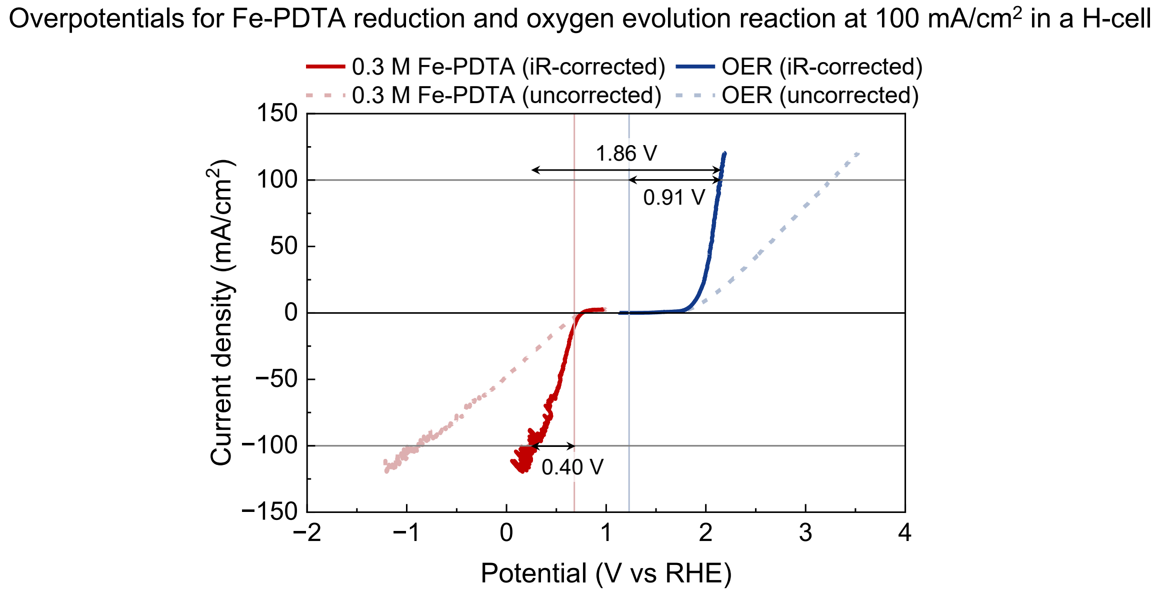

Figure S11. Linear-sweep voltammetry (LSV) curves of the anodic oxygen evolution reaction (OER) and cathodic reduction of 0.3 M Fe-PDTA at a scan rate of 1 mV/s in an H-cell using a three-electrode configuration with 1000 rpm stirring using 1.5 cm magnetic stir bar. The solution resistance was measured by electrochemical impedance spectroscopy, and the LSV curves were corrected using 95% iR compensation. The vertical red and blue lines indicate the half-wave potential of Fe-PDTA (0.68 V vs RHE) and the theoretical OER potential (1.23 V vs RHE), respectively.

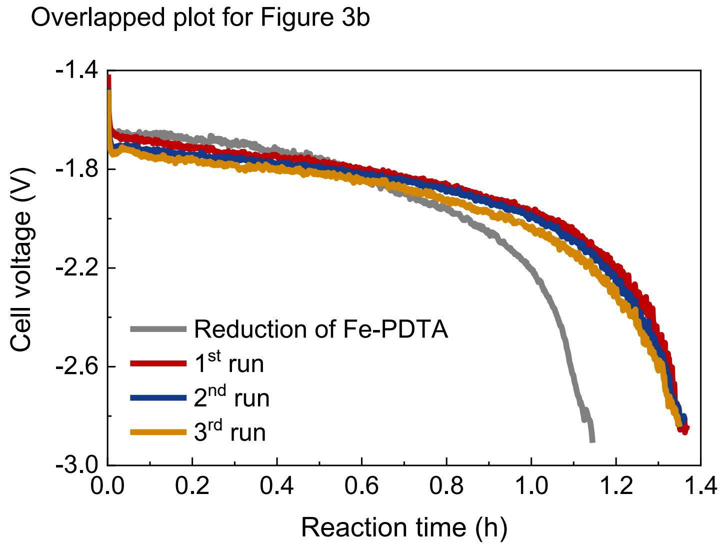

Figure S12. Overlapped chronopotentiometry curves for the closed-loop regeneration reactions.

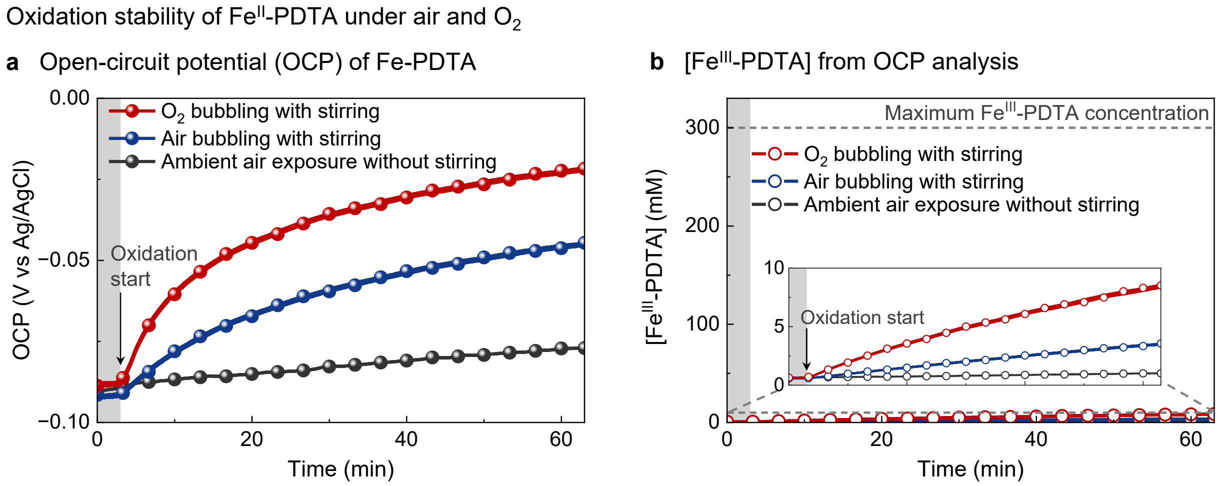

Figure S13. Oxidation stability of Fe^{II}-PDTA under air and O_2. (a) Open-circuit potential (OCP) of 0.3 M Fe^{II}-PDTA solution and (b) Fe^{III}-PDTA concentration as a function of time. The Fe^{III}-PDTA concentration was calculated from the OCP data using the Nernst equation. The solution was first saturated with Ar gas, and OCP monitoring was initiated. After 3 min of OCP measurement, oxidation was initiated by bubbling air or O_2 through the solution. The solution was stirred at 1000 rpm using 1.5 cm stir bar in all experiments, except under the ambient air exposure condition.

7. Characterization of the 100 g scale regenerated $LiFePO_4$

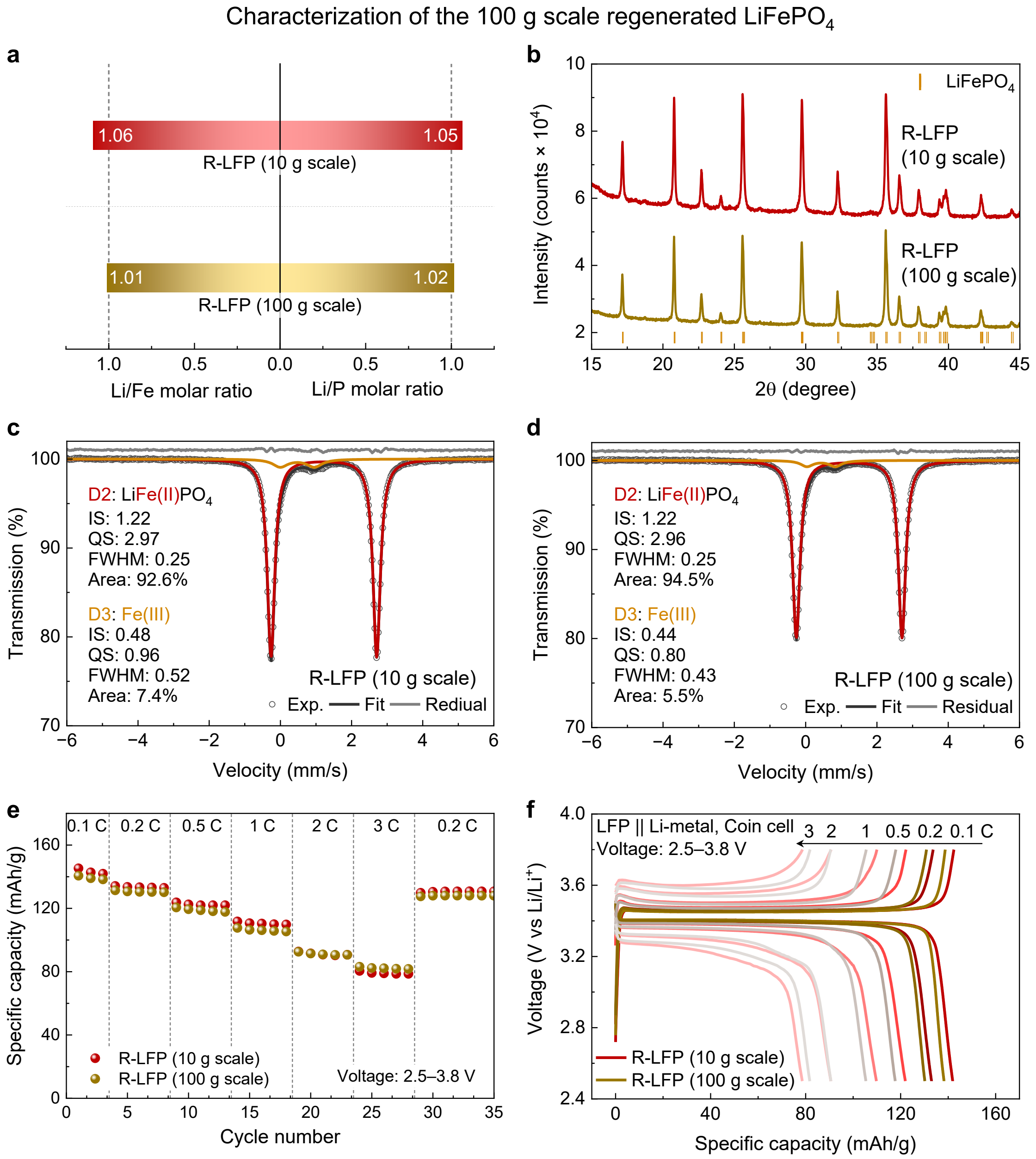

Figure S14. Characterization of the 100 g scale regenerated $LiFePO_4$, R-LFP (100 g scale) and comparison with R-LFP (10 g scale). (a) Li/Fe and Li/P molar ratios measured by inductively coupled plasma optical emission spectrometry, (b) powder X-ray diffraction patterns, ^{57}Fe Mössbauer spectra of (c) R-LFP (10 g scale) and (d) R-LFP (100 g scale) and corresponding fitting parameters, (e) rate performance, and (f) voltage profiles of an LFP/Li-metal coin cell at different C-rates. The D-LFP used in the 100 g scale experiment was isolated from spent LFP cells with similar remaining capacities (40–55%), but it originated from a different batch from that used in the 10 g scale experiments.

8. Fe-PDTA crossover and cycling tests

The crossover of Fe-PDTA mediator from catholyte to anolyte was tested by using a zero-gap flow cell at a constant current density of −20 mA/cm^2 with a flow rate of 50 mL/min. The catholyte consisted of 70 mL of 0.3 M Fe-PDTA, the anolyte contained 20 mL of 1 M borate, and the membrane (Nafion 117) area was 4.84 cm^2. The Fe concentration in the anolyte was monitored over time by ICP-OES analysis. The linear fit corresponds to a permeation rate of 4.43×10^{-13} mol cm^{-2} s^{-1}, which would lead to 130 ppm crossover of Fe-PDTA into the anolyte over 1000 h of operation with the same catholyte volume (200 mL), Fe-PDTA concentration (0.3 M), and membrane area (4.84 cm^2) used in the 100 g scale reaction.

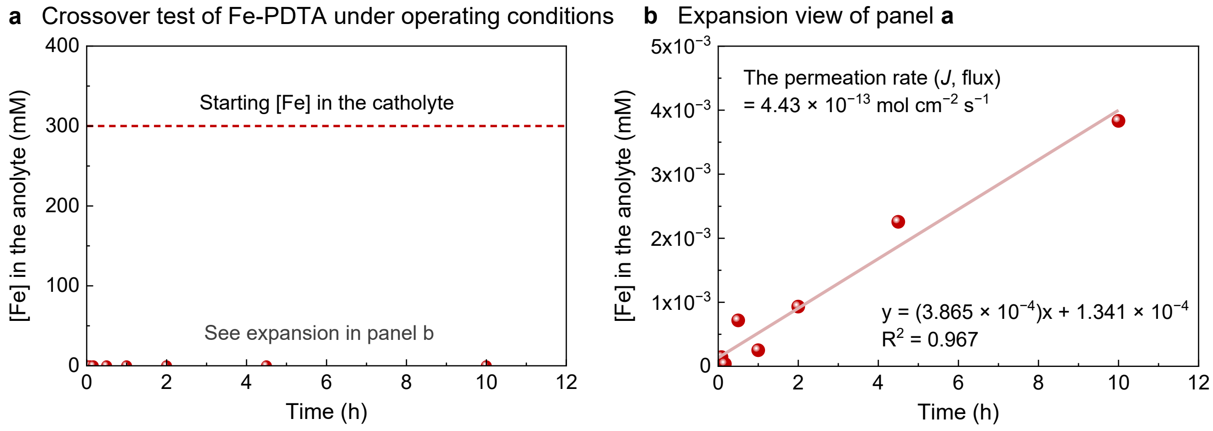

Figure S15. (a) Monitoring of Fe concentration in the anolyte during operation at a constant current of −20 mA/cm^2 with a flow rate of 50 mL/min in a flow-cell configuration; catholyte: 70 mL of 0.3 M Fe-PDTA; anolyte: 20 mL of 1 M borate; membrane area: 4.84 cm^2. (b) Expansion view of panel in panel (a). Aliquots of anolyte were analyzed using inductively coupled plasma-optical emission spectroscopy (ICP-OES). The measured Fe concentrations are plotted on the y-axis.

Galvanostatic cycling tests started with 0.3 M Fe-PDTA in both the catholyte and anolyte with equal amounts of Fe^{III}-PDTA and Fe^{II}-PDTA (i.e., 50% state of charge). To prepare Fe-PDTA at 50% state of charge, 15 mL of 0.3 M Fe^{II}-PDTA was first prepared by electrochemical reduction in a flow cell and then mixed with 15 mL of 0.3 M Fe^{III}-PDTA, yielding 30 mL of 0.3 M Fe^{III}-PDTA/Fe^{II}-PDTA. From this mixture, 10 mL was used as the catholyte (capacity-limiting side) while 20 mL was employed as the anolyte (non-capacity-limiting side). All solutions were degassed by N_2 bubbling prior to the experiments, and cycling tests were conducted in an N_2-purged box. During cycling, the current density was set to 30 mA/cm^2 (electrode area: 4 cm^2), the flow rate was maintained at 50 mL/min, and the reservoirs were stirred at 200 rpm using a 1 cm star-shaped magnetic stir bar. Redox cycling was paused for 1 min after each charge and discharge cycle.

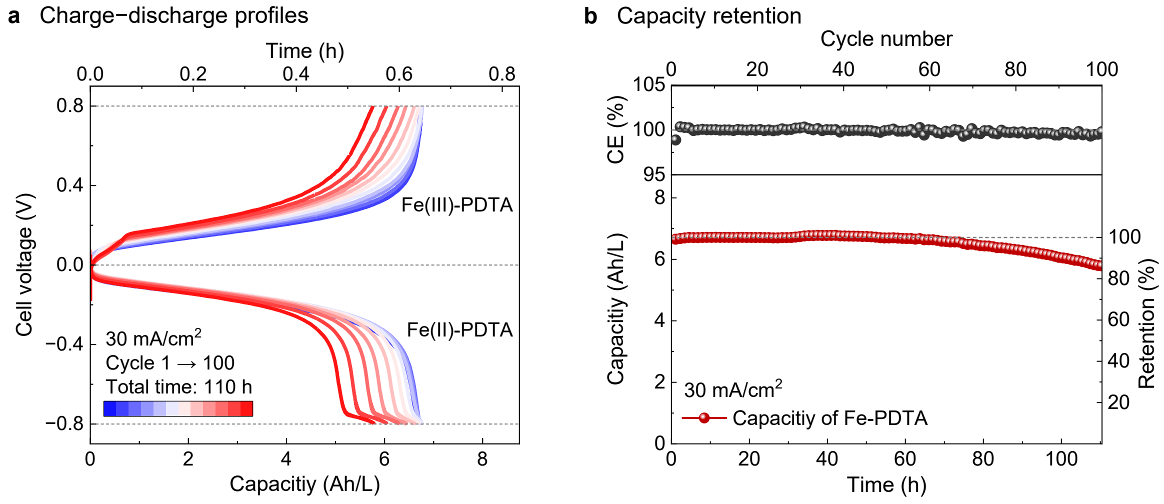

Figure S16. Galvanostatic cycling tests of Fe-PDTA using a symmetric redox flow-battery approach. (a) Charge–discharge profiles for cycles 1–100 within a voltage range −0.8 to 0.8 V, and (b) the corresponding Coulombic efficiency (CE) and capacity retention.

9. Solid-electrolyte interphase species

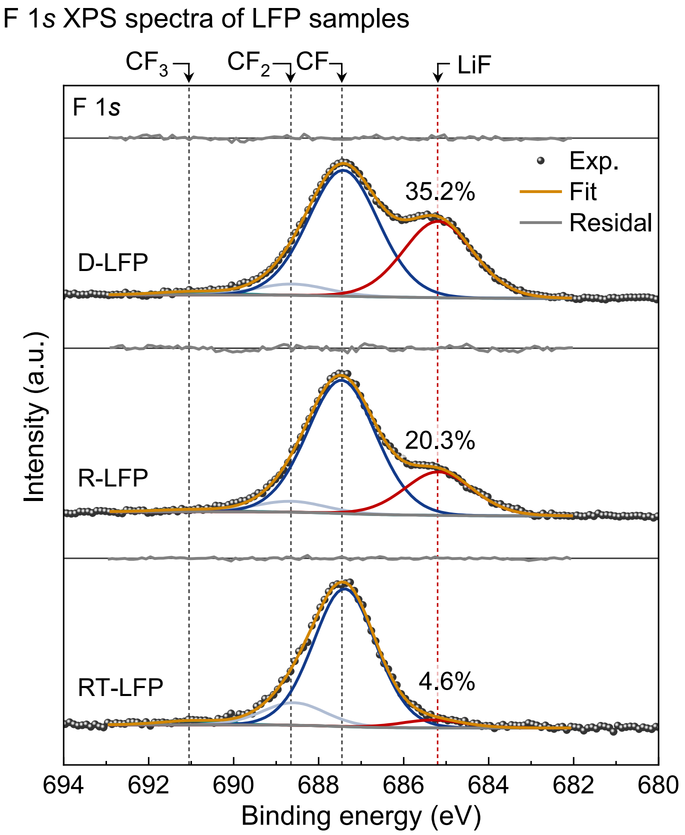

Figure S17. F 1*s* XPS analysis of D-LFP, R-LFP, and RT-LFP, implicating the presence of solid-electrolyte interphase Li species (*i.e.*, LiF) at 685.2 eV.

10. Li–Fe antisite defect analysis using Rietveld refinement of PXRD patterns

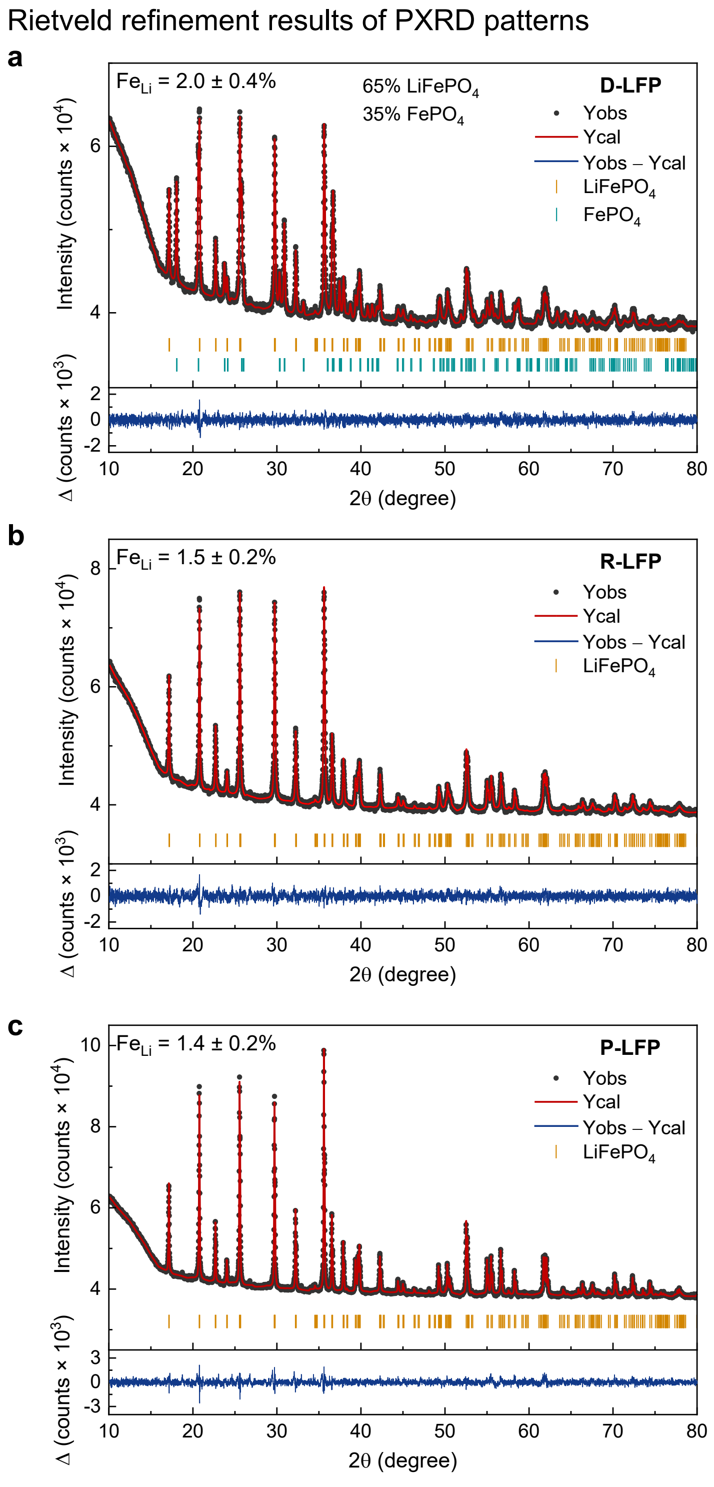

Figure S18. Rietveld refinement results of PXRD patterns. (a) D-LFP, (b) R-LFP, and (c) P-LFP.

Table S1. Rietveld refinement results of D-LFP. Agreement factors: χ^2 = 1.26%, R_{wp} (with background subtraction) = 9.70%.

		$LiFePO_4$, Fraction: 65.1(5)%					$FePO_4$, Fraction: 34.9(4)%)			
Space group		*Pnma* (62)					*Pnma* (62)			
Cell parameters		*a* (Å)	*b* (Å)	*c* (Å)	V (Å^3)		*a* (Å)	*b* (Å)	*c* (Å)	V (Å^3)
		10.3192(2)	6.0022(1)	4.6900(1)	290.487(11)		9.8114(3)	5.7854(1)	4.7799(2)	271.317(14)
Atom	Site	*x*	*y*	*z*	Occ.	Site	*x*	*y*	*z*	Occ.
Li	4a	0	0	0	0.4901(19)	NA				
Fe_{Li}	4a	0	0	0	0.0099(19)	4a	0	0	0	0.0099(19)
Fe	4c	0.2818(2)	0.25	0.9746(6)	0.4901(19)	4c	0.2743(4)	0.25	0.9491(8)	0.4901(19)
Li_{Fe}	4c	0.2818(2)	0.25	0.9746(6)	0.0099(19)	NA				
P_1	4c	0.0944(4)	0.25	0.4147(10)	0.5	4c	0.0966(7)	0.25	0.3986(18)	0.5
O_1	4c	0.0972(10)	0.25	0.7367(16)	0.5	4c	0.1135(12)	0.25	0.7063(28)	0.5
O_2	4c	0.4577(9)	0.25	0.2106(11)	0.5	4c	0.4413(15)	0.25	0.1610(18)	0.5
O_3	8d	0.1674(6)	0.0414(9)	0.2862(10)	1.0	8d	0.1662(9)	0.0360(16)	0.2494(18)	1.0

Table S2. Rietveld refinement results of R-LFP. Agreement factors: χ^2 = 1.38%, R_{wp} (with background subtraction) = 8.60%.

		$LiFePO_4$			
Space group		*Pnma* (62)			
Cell parameters		*a* (Å)	*b* (Å)	*c* (Å)	V (Å^3)
		10.3265(2)	6.0061(1)	4.6923(1)	291.023(9)
Atom	Site	*x*	*y*	*z*	Occ.
Li	4a	0	0	0	0.4923(12)
Fe_{Li}	4a	0	0	0	0.0077(12)
Fe	4c	0.2823(1)	0.25	0.9733(3)	0.4923(12)
Li_{Fe}	4c	0.2823(1)	0.25	0.9733(3)	0.0077(12)
P_1	4c	0.0945(2)	0.25	0.4180(5)	0.5
O_1	4c	0.0980(5)	0.25	0.7438(8)	0.5
O_2	4c	0.4573(5)	0.25	0.2086(6)	0.5
O_3	8d	0.1669(3)	0.0430(5)	0.2847(5)	1.0

Table S3. Rietveld refinement results of P-LFP. Agreement factors: χ^2 = 1.70%, R_{wp} (with background subtraction) = 8.44%.

		$LiFePO_4$			
Space group		*Pnma* (62)			
Cell parameters		*a* (Å)	*b* (Å)	*c* (Å)	V (Å^3)
		10.3207(1)	6.0041(1)	4.6902(1)	290.702(4)
Atom	Site	*x*	*y*	*z*	Occ.
Li	4a	0	0	0	0.4931(11)
Fe_{Li}	4a	0	0	0	0.0069(11)
Fe	4c	0.2820(1)	0.25	0.9755(3)	0.4931(11)
Li_{Fe}	4c	0.2820(1)	0.25	0.9755(3)	0.0069(11)
P_1	4c	0.0947(2)	0.25	0.4186(5)	0.5
O_1	4c	0.0957(5)	0.25	0.7435(8)	0.5
O_2	4c	0.4565(5)	0.25	0.2087(6)	0.5
O_3	8d	0.1665(3)	0.0427(5)	0.2883(5)	1.0

11. ^{57}Fe Mössbauer spectroscopy

The isomer shift (IS) and quadrupole splitting (QS) values for the different Fe environments in **Figure 4h** were adopted from previously reported literature values.[3,8–28] Refined parameters for each quadrupole doublet were the IS, QS, FWHM, and area contribution (A%). According to previously reported parameters for LFP,[3,8–16] the doublets D1, D2, and D3 were assigned as follows: D1 (blue line in **Figures 4i-i** and **S19**) corresponds to Fe^{III} in $FePO_4$ (reported values: IS = 0.39–0.42 mm/s, QS = 1.52–1.56 mm/s).[3,8,9] D2 (red lines in **Figures 4i** and **S19**) corresponds to Fe^{II} in LFP (reported values: IS = 1.20–1.26 mm/s, QS = 2.94–2.99 mm/s).[3,8–16] D3 (orange lines in **Figures 4i** and **S19**) is assigned to Fe^{III} impurities, as the typical IS value for Fe^{III} is 0.4–0.5 mm/s[17] and its broad peak (FWHM: >0.4 mm/s) can arise from amorphous or nanoparticulate phases, surface or structural defects in LFP, and multiple Fe^{III} components (*e.g.*, iron oxides).[11–16,18]

^{57}Fe Mössbauer spectra (295 K) and fitting parameters

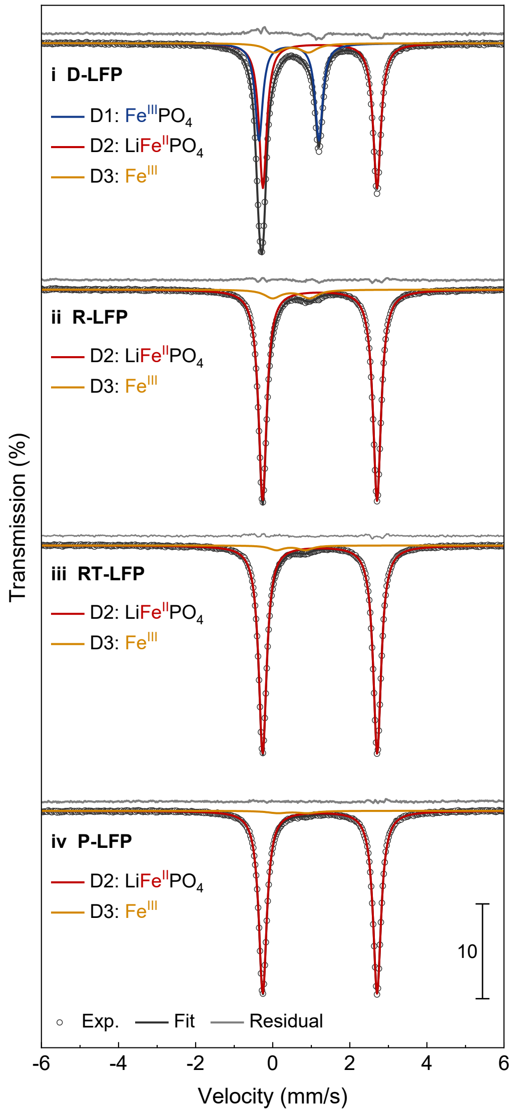

Doublet	IS (mm/s)	QS (mm/s)	FWHM (mm/s)	Area (%)
D1: $Fe^{III}PO_4$	0.42	1.55	0.24	36.7
D1: $LiFe^{II}PO_4$	1.23	2.96	0.24	55.9
D3: Fe^{III}	0.48	0.90	0.53	7.4

Doublet	IS (mm/s)	QS (mm/s)	FWHM (mm/s)	Area (%)
D2: $LiFe^{II}PO_4$	1.22	2.97	0.25	92.6
D3: Fe^{III}	0.48	0.96	0.52	7.4

Doublet	IS (mm/s)	QS (mm/s)	FWHM (mm/s)	Area (%)
D2: $LiFe^{II}PO_4$	1.22	2.97	0.25	96.3
D3: Fe^{III}	0.47	0.75	0.44	3.7

Doublet	IS (mm/s)	QS (mm/s)	FWHM (mm/s)	Area (%)
D2: $LiFe^{II}PO_4$	1.23	2.96	0.25	97.2
D3: Fe^{III}	0.51	0.78	0.63	2.8

Figure S19. ^{57}Fe Mössbauer spectroscopy spectra of (i) D-LFP, (ii) R-LFP, (iii) RT-LFP, (iv) P-LFP, and their fitting parameters.

12. Characterization results of RT-LFP

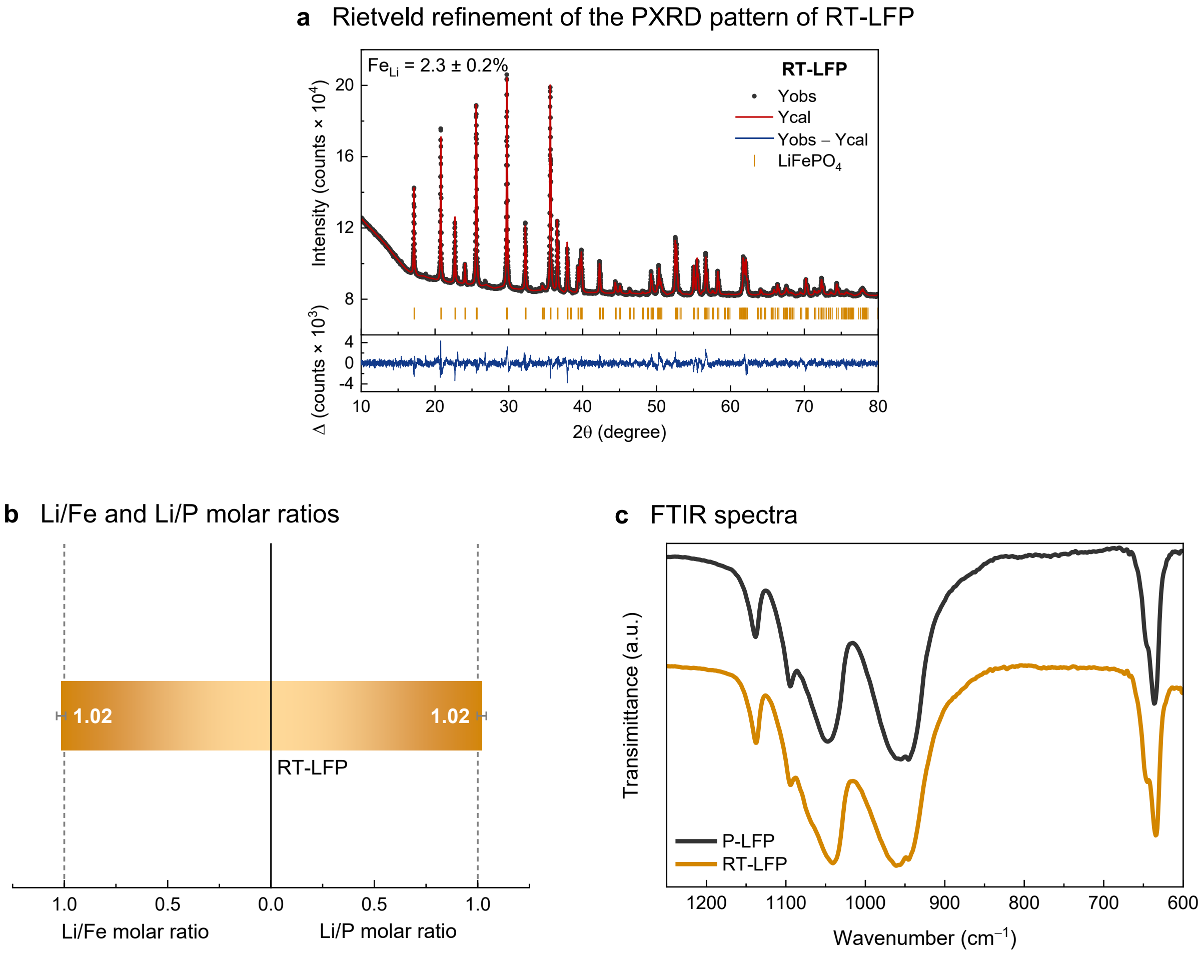

Figure S20. Characterization results of RT-LFP. (a) PXRD pattern, (b) Li/Fe and Li/P molar ratios based on ICP-OES, and (c) FTIR spectra.

Table S4. Rietveld refinement results of RT-LFP. Agreement factors: χ^2 = 2.81%, R_{wp} (with background subtraction) = 6.69%.

		$LiFePO_4$			
Space group		*Pnma* (62)			
Cell parameters		*a* (Å)	*b* (Å)	*c* (Å)	V ($Å^3$)
		10.3289(1)	6.0074(1)	4.6921(1)	291.144(5)
Atom	Site	*x*	*y*	*z*	Occ.
Li	4a	0	0	0	0.4885(9)
Fe_{Li}	4a	0	0	0	0.0115(9)
Fe	4c	0.2828(1)	0.25	0.9703(3)	0.4885(9)
Li_{Fe}	4c	0.2828(1)	0.25	0.9703(3)	0.0115(9)
P_1	4c	0.0923(2)	0.25	0.4161(4)	0.5
O_1	4c	0.0998(4)	0.25	0.7474(7)	0.5
O_2	4c	0.4496(4)	0.25	0.2092(5)	0.5
O_3	8d	0.1625(3)	0.0494(4)	0.2800(4)	1.0

13. Voltage profiles of LiFePO/Li-metal coin cells

Voltage profiles of LFP/Li-metal coin cells at different C-rates

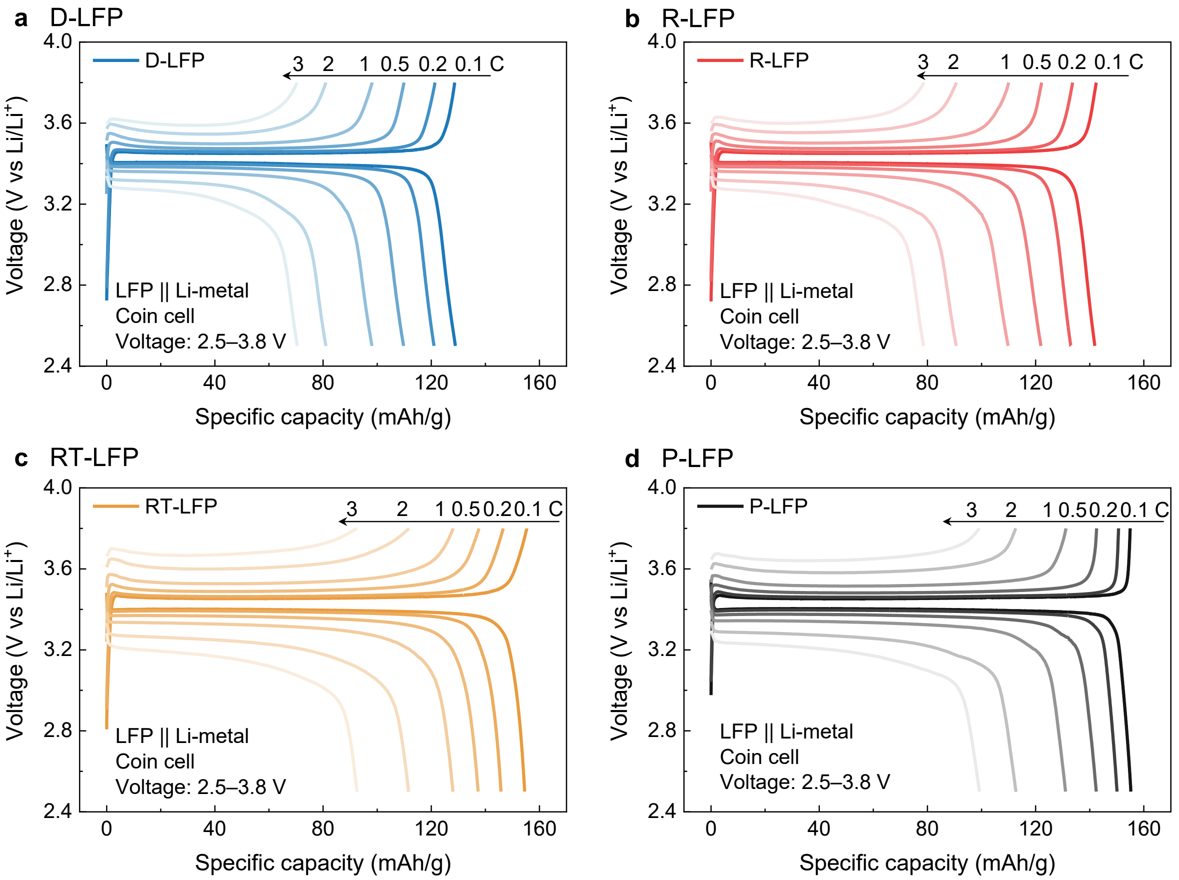

Figure S21. Voltage profiles of LFP/Li-metal coin cells at different C-rates.

14. Techno-economic and environmental analysis

Techno-economic and environmental analysis of the redox-mediated electrochemical direct recycling (denoted as Mediated Echem), mediated chemical lithiation direct recycling,[29–34] pyrometallurgical, and hydrometallurgical recycling processes were conducted using the EverBatt 2020 model developed by Argonne National Laboratory.[35] All recycling processes were evaluated assuming an annual throughput of 10,000 metric tons of spent LFP battery cells at a U.S. geographic location. The electricity price was set to \$0.0902/kWh corresponding to the average U.S. industrial electricity price in September 2025.[36] For the pyrometallurgical and hydrometallurgical recycling processes, the default material and energy input parameters in the EverBatt model were used, except for the updated electricity cost. The cost of relithiation equipment was assumed to be \$4,000,000 for both direct recycling processes, consistent with a previous techno-economic analysis of redox mediator strategy.[30]

Energy input

The energy requirements are summarized in **Table S5**. The energy requirements for diesel and natural gas in direct recycling were adopted from the default values. However, the electricity consumption for the Mediated Echem process was estimated by adding the electrical energy required for the regeneration step to the default electricity demand in the hydrometallurgical process (0.13 MJ/kg cell), due to the similarity in the overall process scheme including low-temperature calcination and several convey equipment (**Figures S25** and **S27**). The electricity consumption for the mediated chemical lithiation was set to 0.13 MJ/kg cell, consistent with the baseline electricity assumption used for the Mediated Echem process. To achieve a throughput of 10,000 metric tons of spent battery cells per year, 1.56 metric ton/h should be processed, assuming 320 operating days per year and 20 hours of actual processing time per day. Based on this processing rate, the electrical energy demand for the electrochemical regeneration step was estimated using the following conditions: 100 mA/cm^2, 1.85 V single cell voltage, 15 stacks, 2.7 m^2 electrode area,[37] and 90% Faradaic efficiency. The electrical energy requirement was calculated as follows:

$$E = Q_{\text{required}} \times \text{V} = Q_{\text{required}} \times n \times \text{V}_{\text{single}} \qquad \text{eq (5)}$$

$$Q_{\text{required}} = \frac{Q_{\text{theoretical}}}{\text{FE}} \qquad \text{eq (6)}$$

$$I_{\text{required}} = \frac{Q_{\text{required}}}{t} \qquad \text{eq (7)}$$

$$n = \frac{I_{\text{required}}}{I_{\text{single}}} = \frac{I_{\text{required}}}{J \times A} \qquad \text{eq (8)}$$

where E is the required energy (J) for processing 1 kg of spent battery, $Q_{\text{theoretical}}$ and Q_{required} are the theoretical and required charges (C) to regenerate 0.382 kg of LFP contained in 1 kg of spent battery, V is the total voltage of the stacked cell (V), n is the number of parallel stacks (n = 15), V_{single} is the voltage per single cell (1.85 V), FE is the Faradaic efficiency (FE = 90%), I_{required} is the required current (A) to process 1 kg of spent battery within 2.3 seconds corresponding to 1.56 metric ton/h, t is the processing time per kg spent battery (t = 2.3 sec), I_{single} is the current (A) for a single cell, and J is the applied current density (A/m^2) corresponding to 100 mA/cm^2, A is the electrode area (2.7 m^2). The resulting electrical energy requirement is 2.52 MJ/kg spent battery cell.

Material input

The material requirements for all recycling processes are summarized in **Table S6**, and the material prices excluding default values are summarized in **Table S7**. For both direct recycling processes, material requirements

(e.g., LiOH, Li metal, mediators, electrolyte, and organic solvents) were calculated based on the assumption of an average delithiation level of 35% for spent LFP and 90% recovery rate for the mediators, electrolyte, and organic solvent. For the Mediated Echem process, 0.27 L/kg cell of 0.3 M Fe-PDTA in 1 M borate electrolyte was required based on 10 g scale experimental conditions and an assumed 90% recovery rate for Fe-PDTA and boric acid. For the mediated chemical lithiation process, 0.42 L/kg cell of 0.1 M benzophenone in dimethoxyethane (DME) was used. The required mediator solution volume was calculated based on the molar quantity of LFP contained in 1 kg of spent battery cell, assuming 35% delithiation level and 90% recovery rate for benzophenone and DME. We note that the assumed 90% recovery rate for Fe-PDTA and boric acid is conservative for the aqueous Mediated Echem process. Specifically, 10% replenishment of 0.3 M Fe-PDTA electrolyte with every cycle (approximately 1 equiv of FeII-PDTA relative to the charge required for LFP regeneration) leaves only 4.1% of the original Fe-PDTA after 75 cycles, even though the remaining original Fe-PDTA retains over 95% of its electrochemical capacity for over 75 cycles (**Figure S16**). Furthermore, the loss of Fe-PDTA through crossover is negligible, corresponding to losses of approximately 10 ppm/day and 0.33%/year based on the permeation rate (4.43×10^{-13} mol cm^{-2} s^{-1} in **Figure S15**), assumed 4,212 L solution volume (2.7 L/kg cell × 1.56 metric tons of cells), 3.267 m^2 membrane area (121% larger than the electrode area), and 15 stacks (see the Energy Input section above). The material cost of Fe-PDTA was estimated by assuming the use of recycled Fe obtained from spent battery casings and the bulk price of ethylenediaminetetraacetic acid (EDTA). Since appropriate environmental impact data for boric acid and EDTA were not available, their environmental impact values associated with energy consumption were approximated using values for phosphoric acid and citric acid, respectively.

It should be noted that direct comparisons among different direct recycling strategies are complicated by intrinsic process differences, including the use of distinct solvents and electron sources. For example, the required use of aprotic organic solvents (*e.g.*, THF, DME, or propylene carbonate) when using lithium metal for LFP regeneration will impact the economic viability of these processes, depending on the efficiency of organic solvent recovery and recycling. Literature precedents are inconsistent on their treatment of this issue, making it difficult to provide a rigorous comparison among different processes. In addition, the use of chemical reductants (*e.g.*, citric acid and ascorbic acid) under aqueous conditions can provide positive profits for LFP recycling, but citric acid dissolves LFP materials to a non-negligible extent under weakly acidic conditions and elevated temperatures.[38,39] These factors should be considered in future rigorous evaluations comparing different direct recycling processes.

In **Figure 6**, pyrometallurgical and hydrometallurgical methods recover only copper, aluminum, graphite, or Li_2CO_3 from spent LFP batteries, and the revenues from these products are insufficient to offset their high capital and chemical costs, resulting in negative profits (−\$2.79 and −\$0.35/kg cell, respectively). In contrast, both direct recycling processes (the Mediated Echem and the mediated chemical lithiation) achieve positive profits of \$3.01 and \$2.38/kg cell, respectively, by producing high-value regenerated LFP (**Figure S22**). The higher profit achieved by the Mediated Echem process arises from the use of LiOH (\$0.32/kg cell) and electricity (\$0.075/kg cell) under aqueous conditions instead of the use of Li metal (\$0.63/kg cell) and organic solvent (\$0.36/kg cell) required in the mediated chemical lithiation (**Figure 6b** and **S22b**). The Mediated Echem process requires additional electricity input, but electricity is a cost-effective utility (\$0.075/kg cell), which is included in the utilities category in **Figure 6b**.

Environmental impact analysis shows that both direct recycling processes exhibit lower or comparable values compared to metallurgical processes (**Figure S23**). Organic solvents dominate the energy consumption and greenhouse gas (GHG) emissions (82% and 43%, respectively) in the mediated chemical lithiation, whereas electricity is the primary contributor (60% and 40%, respectively) in the Mediated Echem process. Environmental impacts can be further reduced by lowering the electrochemical cell voltage through OER optimization (**Figure S24**). However, voltage reduction has a limited impact on total cost, indicating that increasing current density and reaction rate to enable downsizing of electrochemical equipment is a more effective strategy for cost reduction. These analyses suggest pathways to further improve industrial feasibility by using low-cost Li^+ sources (e.g., black mass or brine) and increasing current density to reduce cost while reducing cell voltage to mitigate environmental impacts.

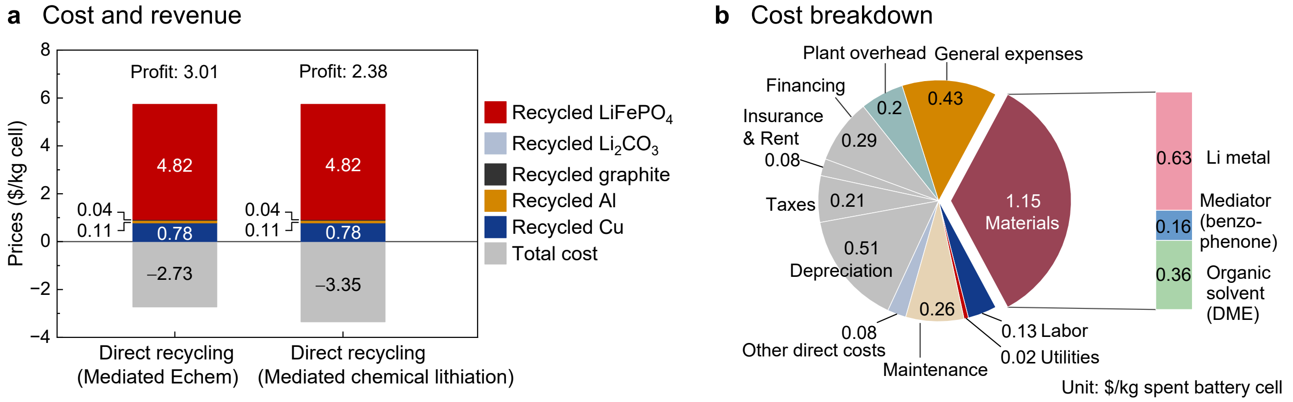

Figure S22. Techno-economic assessment of the mediated chemical lithiation direct recycling. (a) Cost and revenue for recycling 1 kg spent battery cells. (b) Cost breakdown of the mediated chemical lithiation process. The category of other direct costs in the panel includes operating supplies, laboratory charges, patents, and royalties.

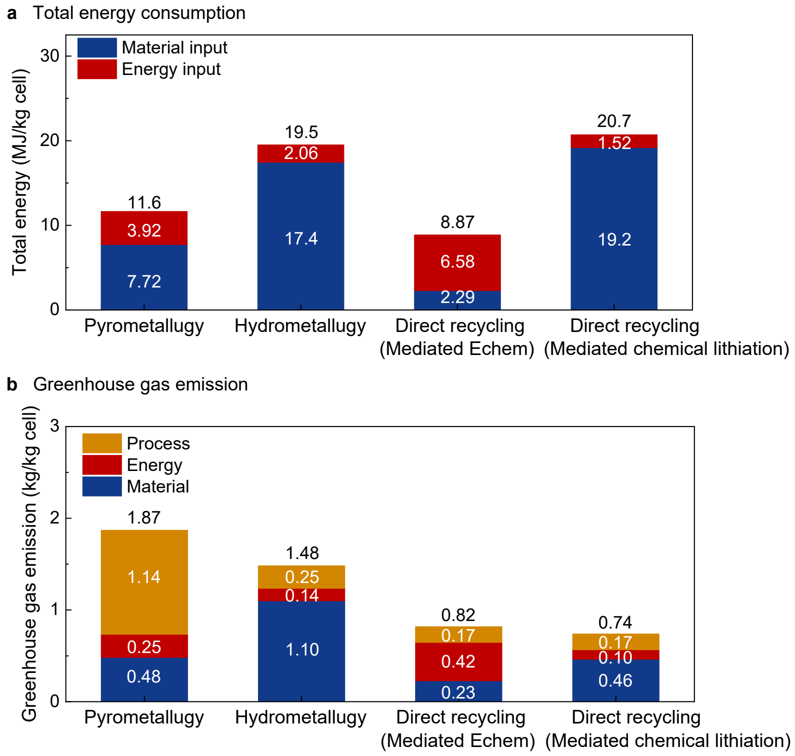

Figure S23. Environmental analysis. (a) Total energy consumption and (b) greenhouse gas emissions for recycling 1 kg spent battery cells.

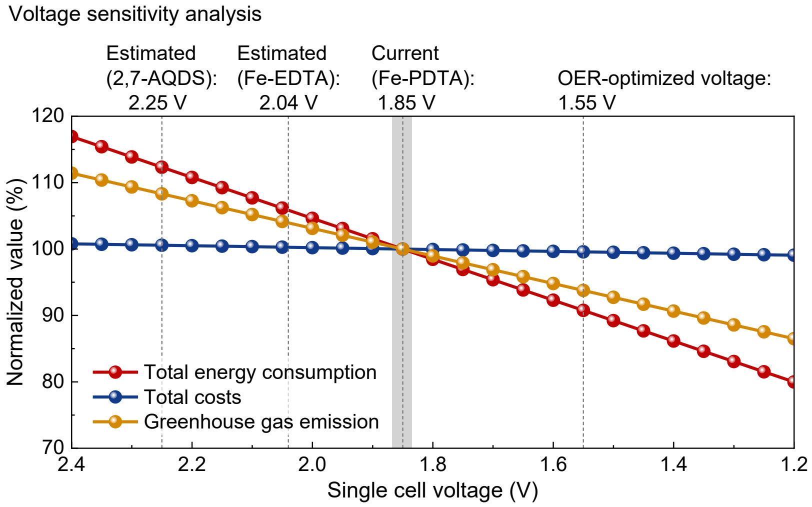

Figure S24. Voltage sensitivity analysis showing the impacts on total energy consumption, total cost, and greenhouse gas emissions. The voltages of 2,7-AQDS and Fe-EDTA were estimated based on their potential differences relative to Fe-PDTA in the CV data (**Figure 2b**); ΔV = 0.4 V for 2,7-AQDS and 0.19 V for Fe-EDTA.

Table S5. Energy requirements (MJ) to recycle 1 kg of spent battery cells.

Energy requirements (MJ/kg cell)	Pyrometallurgy	Hydrometallurgy	Direct recycling (Mediated Echem)	Direct recycling (mediated chemical lithiation)
Diesel	0.60	0.60	0.60	0.60
Natural gas	1.00	1.00	0.50	0.50
Electricity	1.05	0.13	2.65	0.13

Table S6. Material requirements (kg of chemical) to recycle 1 kg of spent battery cells.

Material requirements (kg/kg cell)	Pyrometallurgy	Hydrometallurgy	Direct recycling (Mediated Echem)	Direct recycling (mediated chemical lithiation)
Hydrochloric acid	0.210	0.012	-	-
Hydrogen peroxide	0.060	0.366	-	-
Sodium hydroxide	-	0.313	-	-
Limestone	0.300	-	-	-
Sand	0.150	-	-	-
Sulfuric acid	-	1.078	-	-
Soda ash	-	0.021	-	-
Lithium hydroxide	-	-	0.020	-
EDTA ligand	-	-	0.023	-
Boric acid	-	-	0.017	-
Lithium metal	-	-	-	0.006
Benozophenone	-	-	-	0.008
Dimethoxyethane	-	-	-	0.365

Table S7. Material prices ($/kg of chemical) used in this study that differ from the default values.

Chemical compounds	Prices ($/kg of chemical)	References
Lithium hydroxide (LiOH)	15.74	[40]
Lithium metal	106.3	[41]
EDTA ligand	2.550	[42]
Boric acid	0.810	[43]
Benozophenone	20.81	[32]
Dimethoxyethane	1.000	[30]

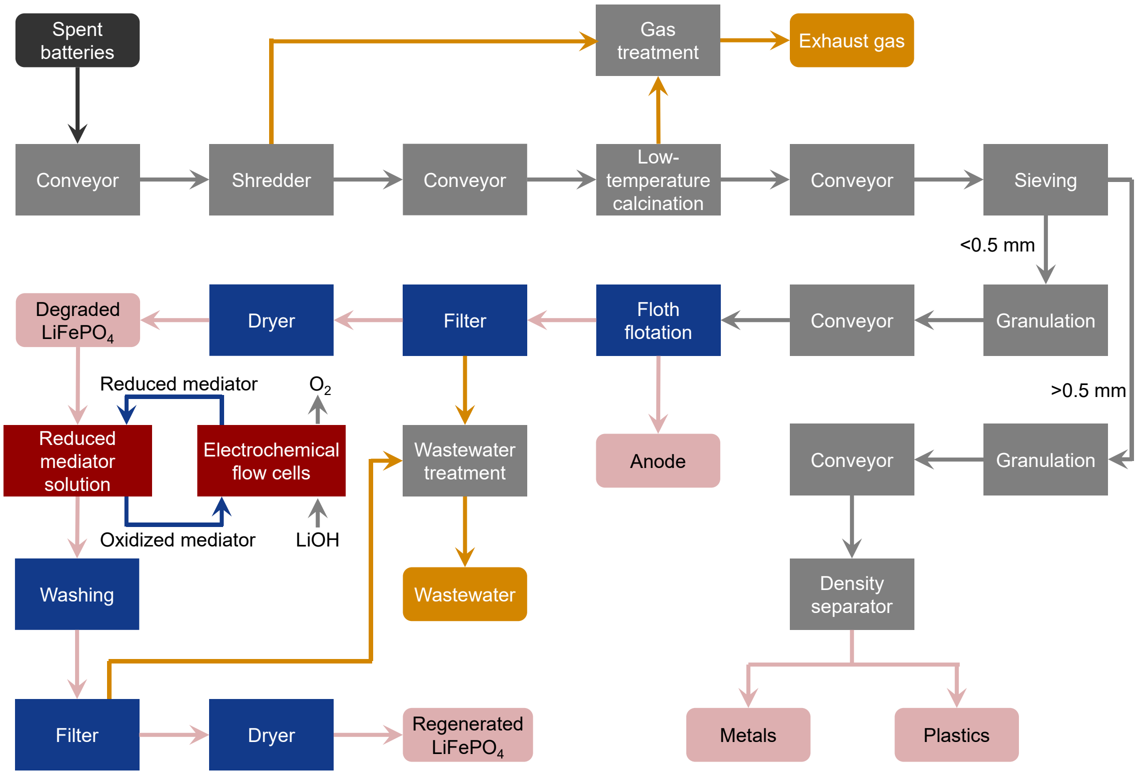

Figure S25. A process diagram of the redox-mediated electrochemical direct recycling (Mediated Echem) process.

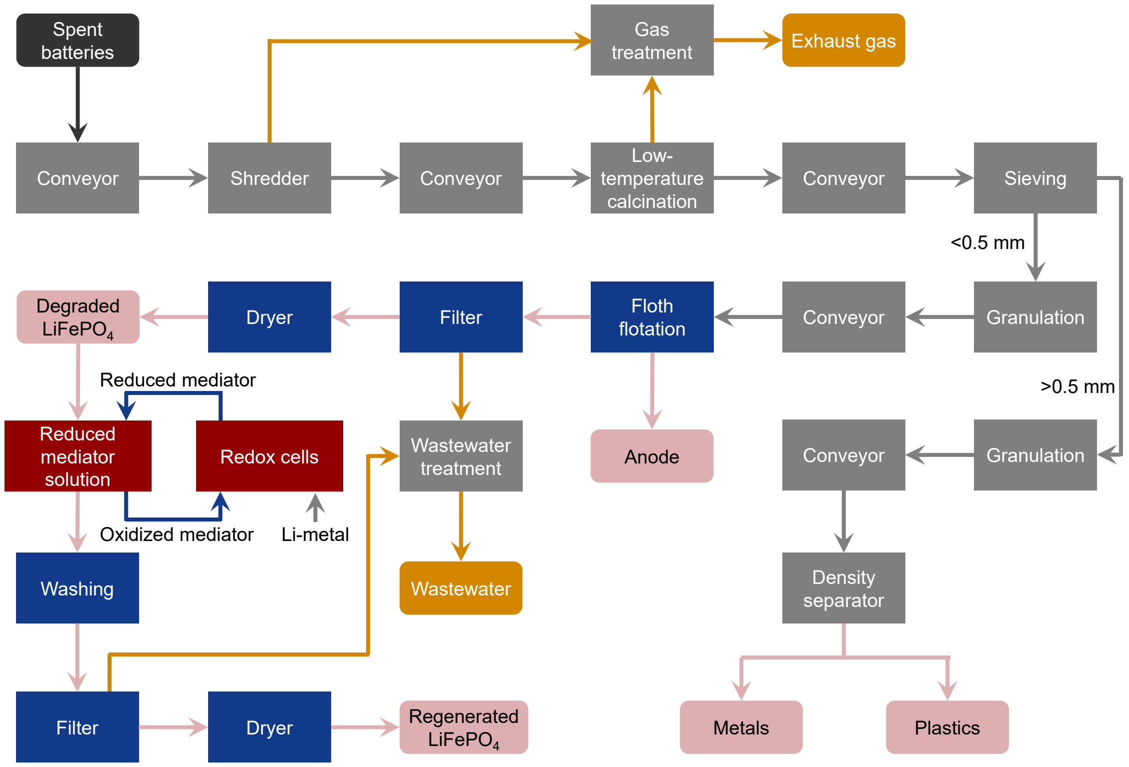

Figure S26. A process diagram of the mediated chemical lithiation direct recycling process.

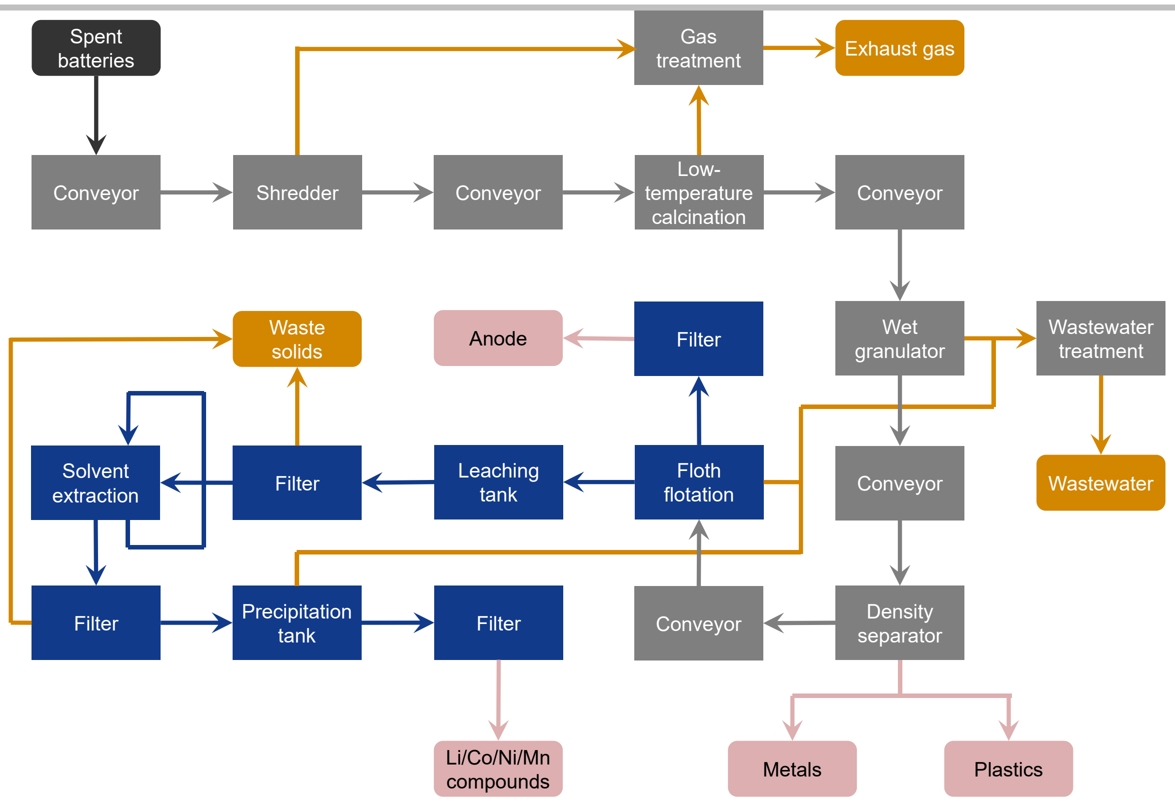

Figure S27. A process diagram of the hydrometallurgical recycling process.

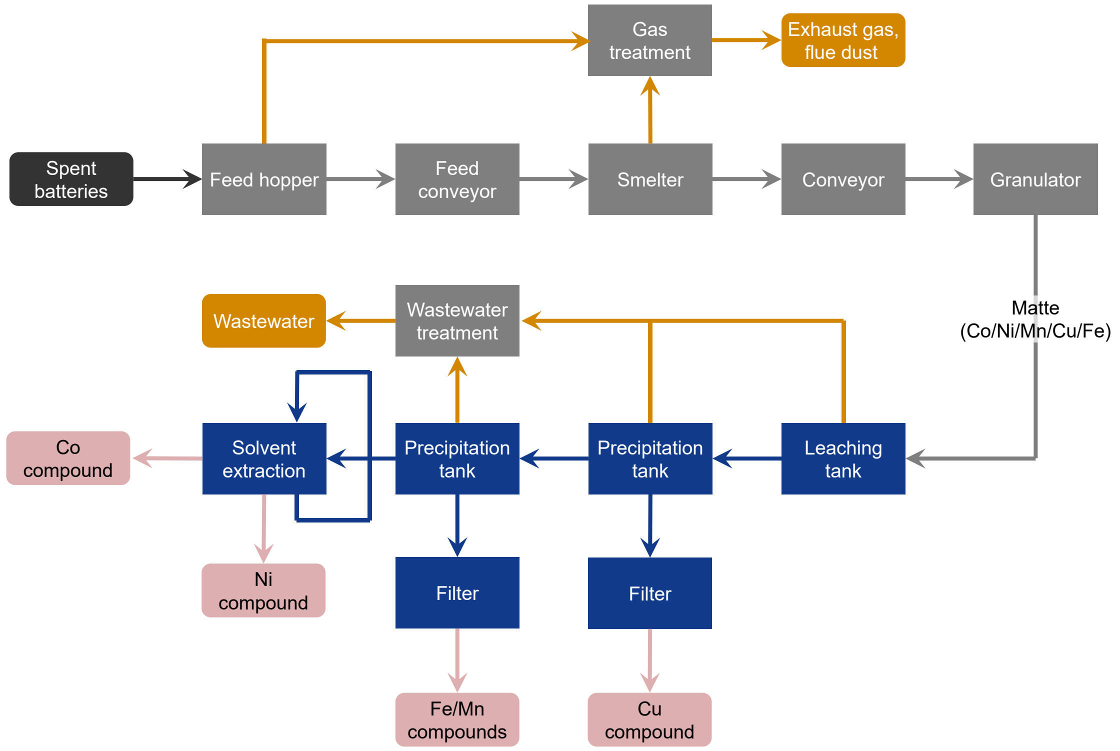

Figure S28. A process diagram of the pyrometallurgical recycling process.

15. Characterization results of Fe-PDTA

a HRMS spectrum of Fe-PDTA

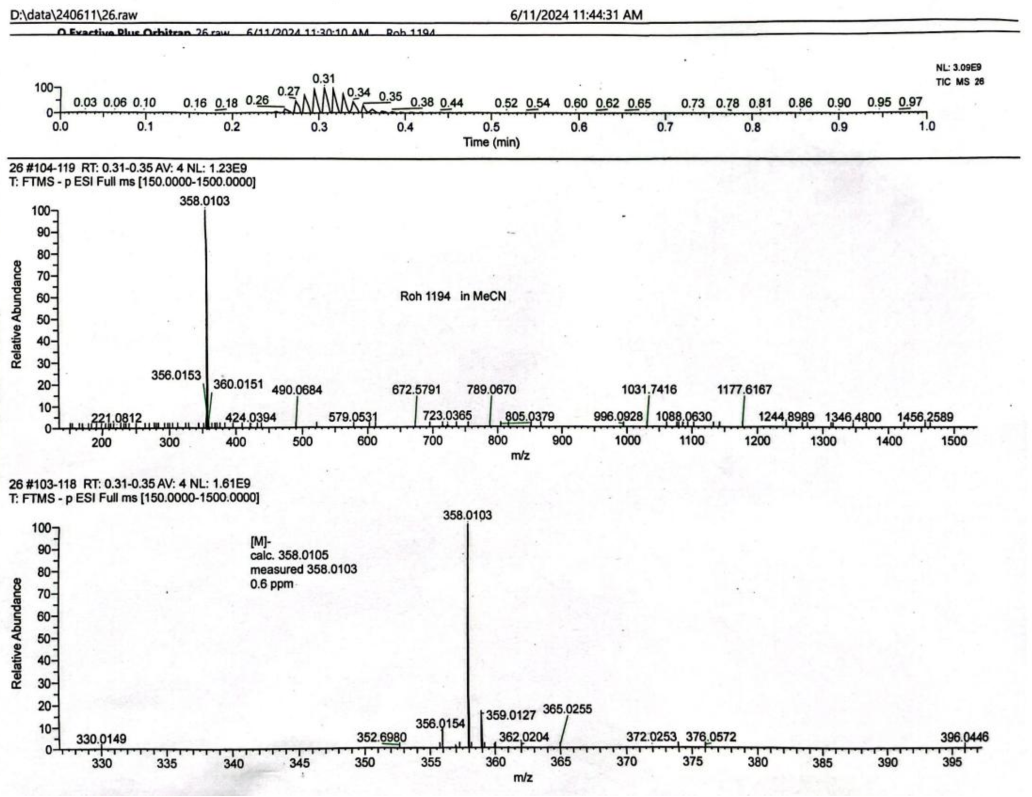

b TGA profile of Fe-PDTA

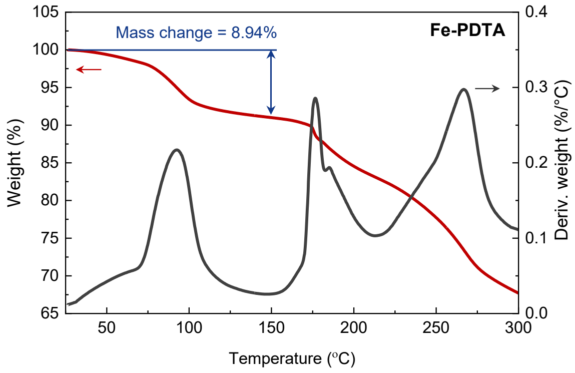

c FTIR spectrum of Fe-PDTA

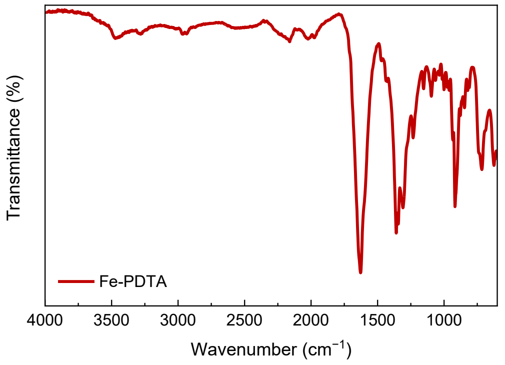

Figure S29. Characterization results of Fe-PDTA. (a) HRMS (ESI) spectra of $[Fe(PDTA)]^-$. (b) TGA profile of Fe-PDTA. TGA was conducted with a heat rate of 10 °C/min under N_2 flow (50 mL/min). (c) FTIR spectrum of Fe-PDTA.

16. Reference

[1] R. Uzal-Varela, F. Lucio-Martínez, A. Nucera, M. Botta, D. Esteban-Gómez, L. Valencia, A. Rodríguez-Rodríguez, C. Platas-Iglesias, "A Systematic Investigation of the NMR Relaxation Properties of Fe(III)-EDTA Derivatives and Their Potential as MRI Contrast Agents" *Inorg. Chem. Front.* **2023**, *10*, 1633–1649.

[2] J. Rodríguez-Carvajal, "Recent Advances in Magnetic Structure Determination by Neutron Powder Diffraction" *Phys. B: Condens. Matter.* **1993**, *192*, 55–69.

[3] A. S. Andersson, B. Kalska, L. Häggström, J. O. Thomas, "Lithium Extraction/Insertion in $LiFePO_4$: an X-ray Diffraction and Mössbauer Spectroscopy Study" *Solid State Ion.* **2000**, *130*, 41–52.

[4] N. Fairley, V. Fernandez, M. Richard-Plouet, C. Guillot-Deudon, J. Walton, E. Smith, D. Flahaut, M. Greiner, M. Biesinger, S. Tougaard, D. Morgan, J. Baltrusaitis, "Systematic and Collaborative Approach to Problem Solving Using X-ray Photoelectron Spectroscopy" *Appl. Surf. Sci.* **2021**, *5*, 100112.

[5] R. Dedryvère, M. Maccario, L. Croguennec, F. Le Cras, C. Delmas, D. Gonbeau, "X-Ray Photoelectron Spectroscopy Investigations of Carbon-Coated Li_xFePO_4 Materials" *Chem. Mater.* **2008**, *20*, 7164–7170.

[6] L. Castro, R. Dedryvère, M. El Khalifi, P.-E. Lippens, J. Bréger, C. Tessier, D. Gonbeau, "The Spin-Polarized Electronic Structure of $LiFePO_4$ and $FePO_4$ Evidenced by in-Lab XPS" *J. Phys. Chem. C* **2010**, *114*, 17995–18000.

[7] H. P. Gunnlaugsson, "Spreadsheet Based Analysis of Mössbauer Spectra" *Hyperfine Interact.* **2016**, *237*, 79.

[8] L. Aldon, A. Perea, M. Womes, C. M. Ionica-Bousquet, J.-C. Jumas, "Determination of the Lamb-Mössbauer Factors of $LiFePO_4$ and $FePO_4$ for Electrochemical *in situ* and *operando* Measurements in Li-Ion Batteries" *J. Solid State Chem.* **2010**, *183*, 218–222.

[9] A. Perea, M. T. Sougrati, C. M. Ionica-Bousquet, B. Fraisse, C. Tessier, L. Aldon, J.-C. Jumas, "*Operando* ^{57}Fe Mössbauer and XRD Investigation of $Li_xMn_yFe_{1-y}PO_4$/C Composites (y = 0; 0.25)" *RSC Adv.* **2012**, *2*, 2080–2086.

[10] D. X. Gouveia, V. Lemos, J. A. C. de Paiva, A. G. Souza Filho, J. Mendes Filho, S. M. Lala, L. A. Montoro, J. M. Rosolen, "Spectroscopic Studies of Li_xFePO_4 and $Li_xM_{0.03}Fe_{0.97}PO_4$ (M=Cr,Cu,Al,Ti)" *Phys. Rev. B* **2005**, *72*, 024105.

[11] X. Xia, Z. Wang, L. Chen, "Regeneration and Characterization of Air-Oxidized $LiFePO_4$" *Electrochem. Commun.* **2008**, *10*, 1442–1444.

[12] N. Dupré, J.-F. Martin, J. Degryse, V. Fernandez, P. Soudan, D. Guyomard, "Aging of the $LiFePO_4$ Positive Electrode Interface in Electrolyte" *J. Power Sources* **2010**, *195*, 7415–7425.

[13] A. Yamada, S. C. Chung, K. Hinokuma, "Optimized $LiFePO_4$ for Lithium Battery Cathodes" *J. Electrochem. Soc.* **2001**, *148*, A224.

[14] R. Amisse, M. T. Sougrati, L. Stievano, C. Davoisne, G. Dražič, B. Budič, R. Dominko, C. Masquelier, "Singular Structural and Electrochemical Properties in Highly Defective $LiFePO_4$ Powders" *Chem. Mater.* **2015**, *27*, 4261–4273.

[15] A. A. M. Prince, S. Mylswamy, T. S. Chan, R. S. Liu, B. Hannoyer, M. Jean, C. H. Shen, S. M. Huang, J. F. Lee, G. X. Wang, "Investigation of Fe Valence in $LiFePO_4$ by Mössbauer and XANES Spectroscopic Techniques" *Solid State Commun.* **2004**, *132*, 455–458.

[16] M. Maccario, L. Croguennec, A. Wattiaux, E. Suard, F. Le Cras, C. Delmas, "C-containing $LiFePO_4$ Materials — Part I: Mechano-Chemical Synthesis and Structural Characterization" *Solid State Ion.* **2008**, *179*, 2020–2026.

[17] F. Menil, "Systematic Trends of the ^{57}Fe Mössbauer Isomer Shifts in (FeO_n) and (FeF_n) Polyhedra. Evidence of a New Correlation Between the Isomer Shift and the Inductive Effect of the Competing Bond T–X (→ Fe) (Where X is O or F and T Any Element with a Formal Positive Charge)" *J. Phys. Chem. Solids* **1985**, *46*, 763–789.

[18] L. Machala, R. Zboril, A. Gedanken, "Amorphous Iron(III) Oxide–A Review" *J. Phys. Chem. B* **2007**, *111*, 4003–4018.

[19] H. J. Tan, J. L. Dodd, B. Fultz, "Mössbauer Spectrometry Study of Thermally-Activated Electronic Processes in Li_xFePO_4" *J. Phys. Chem. C* **2009**, *113*, 2526–2531.

[20] S. Okada, T. Yamamoto, Y. Okazaki, J. Yamaki, M. Tokunaga, T. Nishida, "Cathode Properties of Amorphous and Crystalline $FePO_4$" *J. Power Sources* **2005**, *146*, 570–574.

[21] D. Y. W. Yu, K. Donoue, T. Kadohata, T. Murata, S. Matsuta, S. Fujitani, "Impurities in $LiFePO_4$ and Their Influence on Material Characteristics" *J. Electrochem. Soc.* **2008**, *155*, A526.

[22] M. Cuisinier, J.-F. Martin, N. Dupré, R. Kanno, D. Guyomard, "Elucidating the $LiFePO_4$ Air Aging Mechanism to Predict its Electrochemical Performance" *J. Mater. Chem.* **2011**, *21*, 18575–18583.

[23] J. F. Martin, A. Yamada, G. Kobayashi, S. Nishimura, R. Kanno, D. Guyomard, N. Dupré, "Air Exposure Effect on $LiFePO_4$" *Electrochem. Solid-State Lett.* **2007**, *11*, A12.

[24] W. Porcher, P. Moreau, B. Lestriez, S. Jouanneau, F. Le Cras, D. Guyomard, "Stability of $LiFePO_4$ in Water and Consequence on the Li Battery Behaviour" *Ionics* **2008**, *14*, 583–587.

[25] S. Boyanov, M. Womes, L. Monconduit, D. Zitoun, "Mössbauer Spectroscopy and Magnetic Measurements As Complementary Techniques for the Phase Analysis of FeP Electrodes Cycling in Li-Ion Batteries" *Chem. Mater.* **2009**, *21*, 3684–3692.

[26] M. Tabuchi, S. Tsutsui, C. Masquelier, R. Kanno, K. Ado, I. Matsubara, S. Nasu, H. Kageyama, "Effect of Cation Arrangement on the Magnetic Properties of Lithium Ferrites ($LiFeO_2$) Prepared by Hydrothermal Reaction and Post-annealing Method" *J. Solid State Chem.* **1998**, *140*, 159–167.

[27] A. Goñi, L. Lezama, N. O. Moreno, L. Fournès, R. Olazcuaga, G. E. Barberis, T. Rojo, "Spectroscopic and Magnetic Properties of α-$Li_3Fe_2(PO_4)_3$: A Two-Sublattice Ferrimagnet" *Chem. Mater.* **2000**, *12*, 62–66.

[28] Yu. V. Baldokhin, I. P. Suzdalev, V. E. Prusakov, D. A. Burnazyan, V. P. Korneev, L. V. Kovalenko, G. E. Folmanis, "A Study of Nanostructures Formed in the Hydrogen Reduction of $Fe(OH)_3$" *Russ. J. Phys. Chem. B* **2012**, *6*, 81–88.

[29] C. Wu, J. Hu, L. Ye, Z. Su, X. Fang, X. Zhu, L. Zhuang, X. Ai, H. Yang, J. Qian, "Direct Regeneration of Spent Li-Ion Battery Cathodes via Chemical Relithiation Reaction" *ACS Sustainable Chem. Eng.* **2021**, *9*, 16384–16393.

[30] K. Park, J. Yu, J. Coyle, Q. Dai, S. Frisco, M. Zhou, A. Burrell, "Direct Cathode Recycling of End-Of-Life Li-Ion Batteries Enabled by Redox Mediation" *ACS Sustainable Chem. Eng.* **2021**, *9*, 8214–8221.

[31] S. Ko, J. Choi, J. Hong, C. Kim, U. Hwang, M. Kwon, G. Lim, S. S. Sohn, J. Jang, U. Lee, C. B. Park, M. Lee, "Thermodynamically Controlled Chemical Regeneration of Spent Battery Cathodes Using Recyclable Electron Donors Under Ambient Conditions" *Energy Environ. Sci.* **2024**, *17*, 4064–4077.

[32] J. Ren, X. Wang, Z. Chang, J. Zhu, Q. Zhang, M. Xu, P. Chai, C. Wu, K. Zhang, X. Ai, J. Qian, "Aromatic Ketone-Mediated Two-Electron Lithiation for Rapid and Room-Temperature Regeneration of Spent $LiFePO_4$ Cathodes" *J. Am. Chem. Soc.* **2025**, *147*, 44972–44983.

[33] M. Xu, C. Wu, F. Zhang, Y. Zhang, J. Ren, C. Zhang, X. Wang, L. Xiao, O. Fontaine, J. Qian, "Potential Regulation Strategy Enables Ferrocene as p-Type Redox Mediator for Direct Regeneration of Spent $LiFePO_4$ Cathode" *Energy Storage Mater.* **2024**, *71*, 103611.

[34] M. Xu, C. Wu, L. Ye, Y. Zhang, C. Zhang, J. Hu, R. Tan, D. Gu, X. Wang, O. Fontaine, C. Zhan, L. Zhuang, X. Ai, J. Qian, "Direct Regeneration of Spent $LiCoO_2$ Black Mass Based on Fluorenone-Mediated Lithium Supplementation and Energy-Saving Structural Restoration" *Adv. Energy Mater.* **2024**, *14*, 2401197.

[35] Q. Dai, J. Spangenberger, S. Ahmed, L. Gaines, J. C. Kelly, M. Wang, *EverBatt: A Closed-loop Battery Recycling Cost and Environmental Impacts Model*, Argonne National Laboratory (ANL), Argonne, IL (United States), **2019**.

[36] "The average U.S. industrial electricity price in September 2025," can be found under https://www.eia.gov/electricity/monthly/epm_table_grapher.php?t=epmt_5_6_a(accessed 15 December 2025), **2025**.

[37] C. Minke, U. Kunz, T. Turek, "Techno-Economic Assessment of Novel Vanadium Redox Flow Batteries with Large-Area Cells" *J. Power Sources* **2017**, *361*, 105–114.

[38] P. Xu, Q. Dai, H. Gao, H. Liu, M. Zhang, M. Li, Y. Chen, K. An, Y. S. Meng, P. Liu, Y. Li, J. S. Spangenberger, L. Gaines, J. Lu, Z. Chen, "Efficient Direct Recycling of Lithium-Ion Battery Cathodes by Targeted Healing" *Joule* **2020**, *4*, 2609–2626.

[39] J. Tang, H. Qu, C. Sun, X. Xiao, H. Ji, J. Wang, J. Li, G. Ji, X. Zhang, H.-M. Cheng, G. Zhou, "A Universal Solution for Direct Regeneration of Spent Lithium Iron Phosphate" *Adv. Mater.* **2025**, *37*, 2420238.

[40] "The bulk price of lithium hydroxide," can be found under https://www.metal.com/en/prices/201102250281(accessed 3 January 2026), **2026**.

[41] "The bulk price of lithium metal," can be found under https://www.metal.com/en/prices/202304250002(accessed 3 January 2026), **2026**.

[42] "The bulk price of ethylenediaminetetraacetic acid (EDTA)," can be found under https://dir.tridge.com/prices/disodium-ethylenediaminetetraacetate(accessed 15 December 2025), **2025**.

[43] "The bulk price of boric acid," can be found under https://businessanalytiq.com/procurementanalytics/index/boric-acid-price-index/(accessed 9 December 2025), **2025**.
